# Guest-dependent directional complexation based on triptycene derived oxacalixarene: formation of oriented rotaxanes†
†Electronic supplementary information (ESI) available: Further details of synthesis, characterization, ^1^H and 2D NMR experiments and computational modeling. CCDC 1009829, 1009830, and 1419743. For ESI and crystallographic data in CIF or other electronic format see DOI: 10.1039/c5sc03511b


**DOI:** 10.1039/c5sc03511b

**Published:** 2015-10-08

**Authors:** Han-Xiao Wang, Zheng Meng, Jun-Feng Xiang, Yu-Xiang Xia, Yihua Sun, Shu-Zhen Hu, Hui Chen, Jiannian Yao, Chuan-Feng Chen

**Affiliations:** a Beijing National Laboratory for Molecular Sciences, CAS Key Laboratory of Molecular Recognition and Function, Institute of Chemistry, Chinese Academy of Sciences, Beijing 100190, China. Email: cchen@iccas.ac.cn; b CAS Key Laboratory of Photochemistry, Institute of Chemistry, Chinese Academy of Sciences, Beijing 100190, China. Email: chenh@iccas.ac.cn; c University of Chinese Academy of Sciences, Beijing 100049, China

## Abstract

The manipulation of supramolecular devices to carry out sophisticated and programmed tasks is bound up with the spatial allocation of their components, especially the threading direction of the guest, which controls the host–guest orientation in the device. However, insights are needed to probe more possibilities for steering the threading direction. We have developed a new system consisting of a three-dimensional nonsymmetric oxacalixarene (**H**) with a fixed comformation and (bi)pyridinium salts (**G1–G3**), in which we found that based on the intrinsic discrepancies between the two semi-cavities of **H**, the electron densities of the axles greatly affect the threading direction. This was unequivocally demonstrated by NMR spectra and single crystal structures. With elaborate design, unidirectional threading was achieved, resulting in an oriented rotaxane. Therefore, we describe a new approach in which the threading direction and final orientation may be finely controlled by adjustment of the structure of the guest.

## Abstract 2

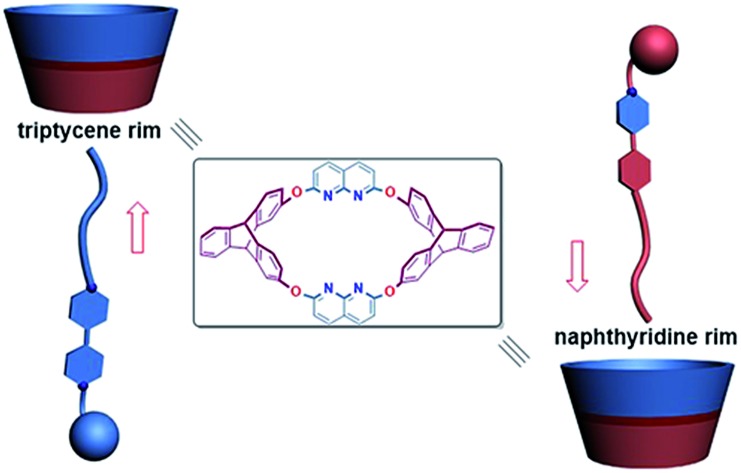
Structural changes in the guest cause inversion of the dominant threading direction in triptycene derived oxacalixarene with different semi-cavities.

## Introduction

During the last two decades, great progress has been made in the synthesis of mechanically interlocked molecules (MIMs) such as rotaxanes and catenanes,^1^ with interest arising not only from their aesthetic appeal^2^ but also their perceived potential to act as sophisticated molecular machines, switches and sensors.^3^ The general approach to constructing (pseudo)-rotaxanes requires a macrocycle and a linear guest as the wheel and the axle, respectively; if neither of them is in the *D*_nh_ point group, two isomers will be obtained *via* the threading process, resulting in different orientations of components in the final MIMs.^4^–^7^ Owing to differences in the spatial arrangement, a pair of orientational isomers may generate quite different properties.^8^ However, the separation of isomers poses a big problem,^4^,^6^,9b so unidirectional threading that leads to an oriented rotaxane is highly appealing. Using cyclodextrin as the wheel, unidirectional threading has been realized due to either kinetic^10^ or thermodynamic^11^ stability, resulting in various oriented rotaxanes. Calixarenes with three-dimensional nonsymmetric structures have provided another candidate to form oriented rotaxanes, based on the formation of endo-cavity complexes with organic cations.^9^,^12^,^13^ Using triphenylureido-calix[6]arene as the host and dialkylviologen salt as the guest, Arduini and coworkers succeeded in synthesizing an oriented pseudorotaxane through a kinetically controlled process.9*a* On that basis, they prepared a pair of isomeric oriented rotaxanes for the first time by reversing the sequence in which two different stopper groups were introduced onto the axle.^4^ In contrast to the kinetically controlled unidirectional threading strategy, Neri and coworkers developed an “endo-alkyl” rule controlling the threading directionality of alkylbenzylammonium axles with calix[6]arenes.^5^ Applying the rule, they constructed stereoisomeric (pseudo)[3]rotaxanes13a,b and an oriented handcuff rotaxane.13*c* Although some important results have been obtained, the formation of oriented rotaxanes based on controllable threading directionality is still emerging as a challenge to be overcome.

**Fig. 1:**
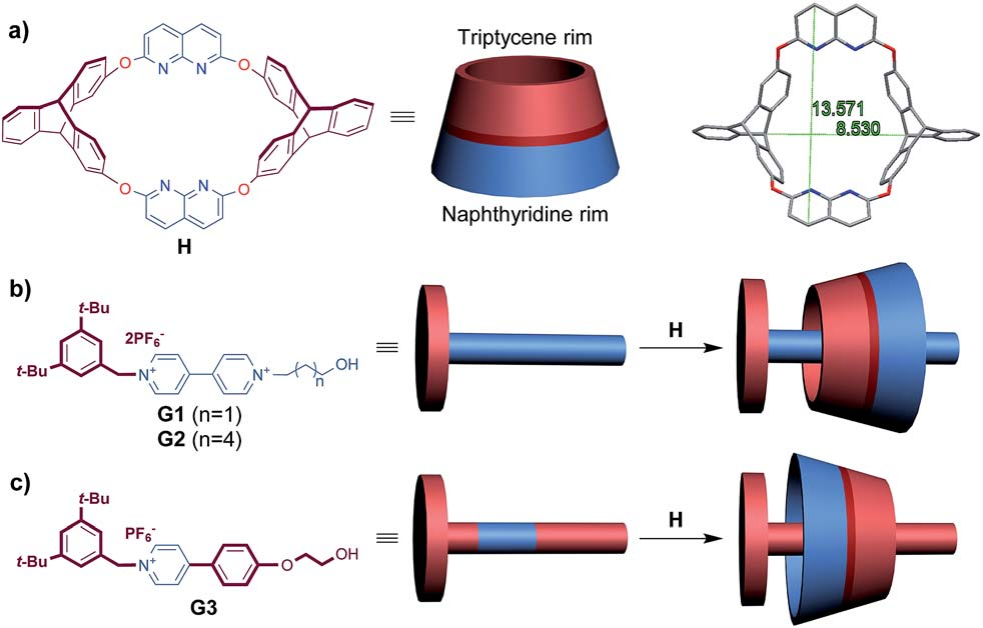
(a) Structure and representation of **H** and its crystal structure,16*a* denoting sizes of the portals on both sides. (b) Structure and representation of guests **G1–G2** and their directional threading into **H**. (c) Structure and representation of guest **G3** and its directional threading into **H**.

Heterocalixarenes^14^ are a kind of calixarenes obtained by replacing conventional methylene bridges with heteroatoms. Introducing bridging heteroatoms makes it possible to finely tune the size, conformation and binding properties of the macrocycle, since heteroatoms can not only adopt different electronic configurations but also form various degrees of conjugation with the neighboring aromatic rings.^15^ Due to the difficulty of forming “through-the-annulus” complexes with linear guests, the utilization of heterocalixarenes as hosts to realize directional threading and construction of oriented rotaxanes is still a rather unexplored field. Previously, we reported that a triptycene-derived oxacalixarene **H**16*a* (Fig. 1a), with an upper semi-cavity encircled by two naphthyridine moieties and a lower semi-cavity encircled by two triptycene moieties, could incorporate various 4,4′-bipyridinium salts to form pseudo-rotaxanes.16*b* We applied this property to the synthesis of rotaxanes based on **H** and symmetric 4,4′-bipyridinium salts. Theoretically, if we mixed **H** with a nonsymmetric axle, a pair of isomeric pseudorotaxanes might be obtained. The crystal structure of **H**16*a* disclosed that the portal of the lower semi-cavity was more crowded than that of the upper one (Fig. 1a, 8.53 Å *versus* 13.57 Å). Moreover, triptycene is known to be more electron-rich than the naphthyridine moiety (the N atoms excluded). Hence, we hypothesized that the discrepancies in size and electron density between the two semi-cavities of **H** might exert different induction effects upon the threading of guests. Herein, we report a system employing **H** as the wheel, in which we can control the dominant threading direction by partially changing the structures of the axles, thus tuning their interaction with **H** in the threading process.

## Results and discussion

### Directional complexation between macrocycle **H** and bipyridinium salts **G1** and **G2** with alkyl chains of different lengths

**Scheme 1:**
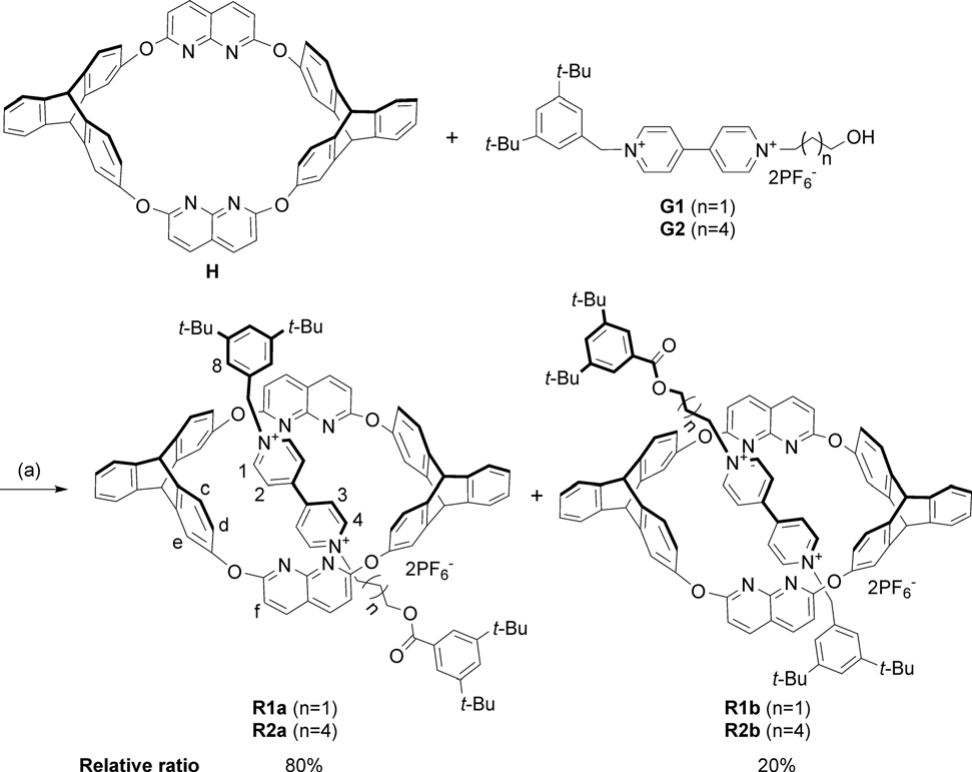
Synthesis of isomeric [2]rotaxanes **R1a–b** and **R2a–b**. Conditions: (a) 3,5-di-*tert*-butylbenzoic anhydride, (*n*-Bu)_3_P, CHCl_3_/CH_3_CN (2 : 1, v/v), r.t.

We initially designed a linear nonsymmetric bipyridinium salt **G1** (Fig. 1b), stoppering one of its ends with a bulky terminal group in order to easily clarify its threading direction into the macrocycle. By comparison of the ^1^H NMR spectra of **H**, **G1**, and a 1 : 1 mixture of the two in CDCl_3_/CD_3_CN solution at room temperature, complexation between the two components was observed (Fig. S31, ESI†). The ^1^H NMR spectrum of the complex always exhibited only one set of signals, regardless of whether the host or the guest was in excess (Fig. S34, ESI†), indicating a fast equilibrium on the NMR timescale. To acquire information on the relative orientation of the two components, we stoppered the pseudorotaxanes to synthesize the corresponding rotaxanes (Scheme 1).

As we demonstrated in our previous work that macrocycle **H** adopted a fixed 1,3-alternate conformation and would not undergo comformational inversion mainly due to the bulky triptycene moieties,^16^ the orientation of the obtained rotaxanes could represent the original threading direction of the axle into the wheel on a statistical basis.9*b* The stoppered products were isolated as a mixture of two orientational isomers, as revealed by both ^1^H NMR spectra and ESI-HRMS. The integrals in the ^1^H NMR spectrum (see Fig. S27†) showed that the relative ratio of the two sets of signals (and thus the amounts of the two isomers, **R1a**‡
‡In this paper, isomers of pseudorotaxanes and rotaxanes are denoted such that a indicates that the corresponding guest is threaded from the triptycene rim, while b indicates that the corresponding guest is threaded from the naphthyridine rim. and **R1b**) was approximately 4 : 1. The ratio calculated from the NMR spectrum revealed that the threading was highly directionally selective, but we could not ascertain the relative orientation of the wheel and axle in the two isomers.

**Fig. 2:**
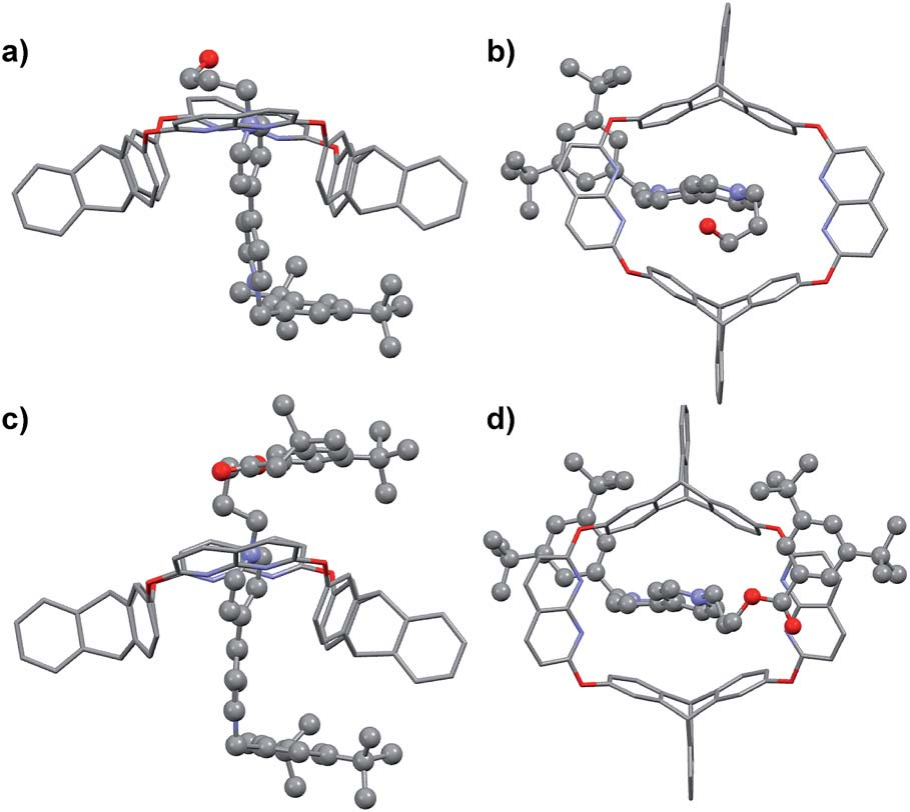
(a) Side and (b) top view of the crystal structure of **H**@**G1**. (c) Side and (d) top view of the crystal structure of [2]rotaxane **R1a**. Hydrogen atoms and PF_6_^–^ counterions are omitted for clarity.

Fortunately, single crystals of both the pseudorotaxane cultivated from the CHCl_3_/CH_3_CN solution of the complex (Fig. 2a and b) and rotaxane **R1a** cultivated from its CHCl_3_ solution (Fig. 2c and d) were obtained. Both crystal structures showed that the axle had threaded into the wheel from the triptycene rim (the lower rim, Fig. 1b).§
§The absence of the pseudorotaxane in which the macrocycle and axle are oriented the other way (**PR1b**) in the crystal might be caused by the difference in the amounts of the two isomeric pseudorotaxanes and the selective crystallization of the dominant one. Weak correlations in the 2D ROESY spectrum of **R1a** (Fig. S22, ESI†) between H_2_ and H_c_, H_2_ and H_d_, and H_8_ and H_c_ were also detected, which indicated the same orientation of the host and guest in **R1a** as was deduced from the crystal structure.

In order to determine which part of the axle might have exerted a crucial impact on the threading direction,9*d* we firstly altered the length of the alkyl chain by replacing the hydroxypropyl group with a hydroxyhexyl group (**G2**, Fig. 1b). We synthesized the corresponding isomeric rotaxanes **R2a** and **R2b** (the ratio was also found to be about 4 : 1, Fig. S28, ESI†) (Scheme 1) and compared the ^1^H NMR spectrum of the major product **R2a** with that of **R1a**. After unambiguous assignment of all signals in the ^1^H NMR spectrum of **R2a** on the basis of 2D NMR experiments (Fig. S23 and S24, ESI†), it was found that all the corresponding hydrogens of the two rotaxanes had almost the same chemical shifts (Fig. S9 and S13, ESI†), indicating identical orientation of the wheel and axle in **R1a** and **R2a**. Thus it could be summarized that the length of the terminal alkyl chain had a negligible influence on the threading direction, at least when the chain was not very long.

### Unidirectional complexation between macrocycle **H** and pyridinium salt **G3**

**Scheme 2:**
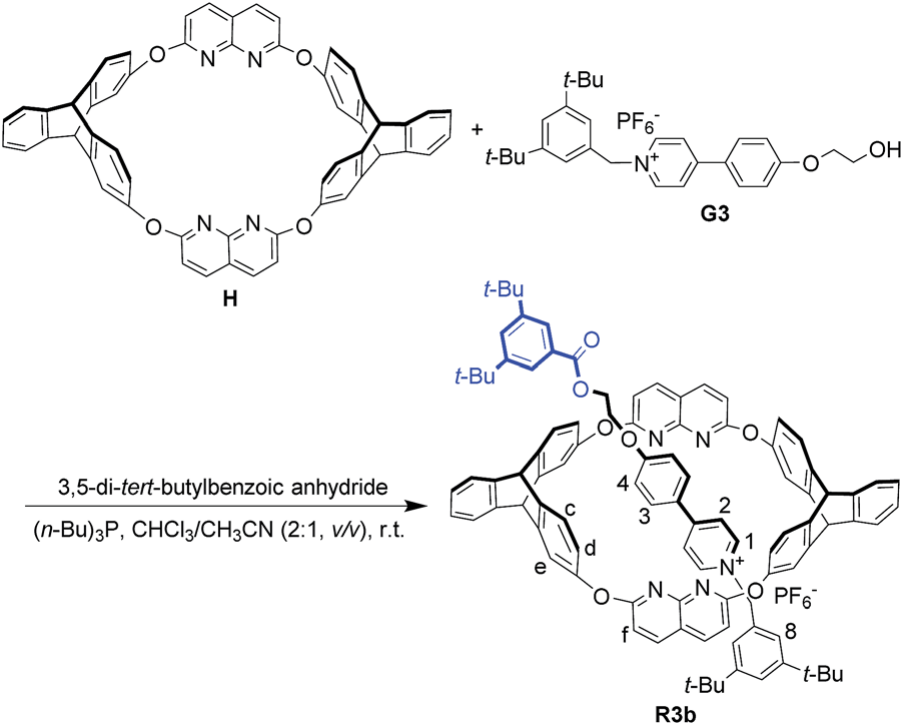
Synthesis of oriented [2]rotaxane **R3b**.

**Fig. 3:**
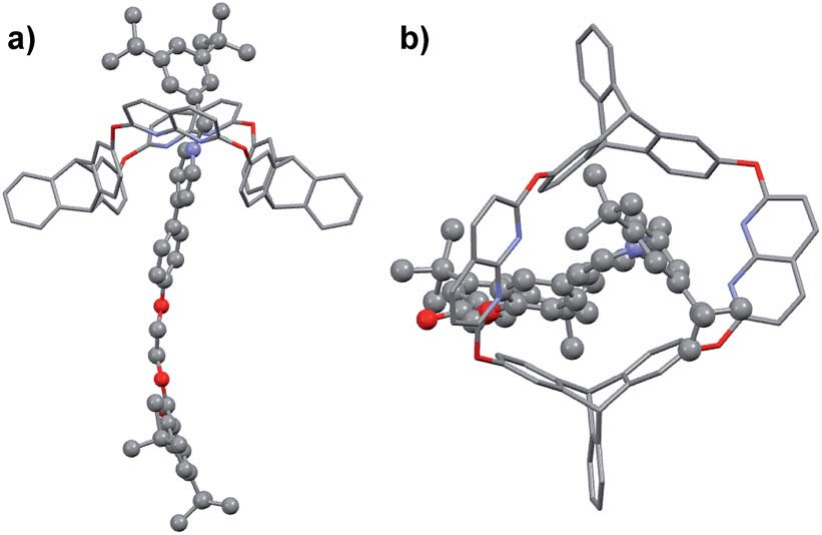
(a) Side and (b) top view of the crystal structure of **R3b**. Hydrogen atoms and PF_6_^–^ counterions are omitted for clarity.

In the dominant threading direction of **G1**, the process was electrostatically favorable but sterically unfavorable. Therefore, to disclose whether electrostatic effects might have played an important part in the directional selectivity, we replaced the terminal pyridyl ring in **G1** with a more electron-rich phenyl ring substituted by an electron-donating alkoxy group (Fig. 1c). Pyridinium salt **G3** was encapsulated by **H** as expected (Fig. S33, ESI†), and similarly, the complex was in fast exchange on the NMR timescale (Fig. S40, ESI†). As the relevant data of the pseudorotaxane provided barely any information about the threading direction, the host–guest complex was stoppered to give the corresponding rotaxane (Scheme 2). To our surprise, the product showed only one set of signals in the ^1^H NMR spectrum (Fig. S19, ESI†), which meant that only one (**R3b**) of the isomeric rotaxanes was actually formed and thus the threading had occurred unidirectionally. To clarify the threading direction of **G3**, we cultivated a single crystal that was suitable for X-ray crystal analysis by slow evaporation of diethyl ether into an acetone solution of **R3b** (Fig. 3). The crystal structure showed that **G3** had threaded into **H** from the naphthyridine rim (Fig. 1c), the direction of which was completely reversed compared with the dominant threading direction of **G1** and **G2**. This agrees well with the 2D ROESY experiment (Fig. S26, ESI†) in which correlations were observed between H_3_ and H_c_/H_d_, H_2_ and H_c_/H_d_, H_8_ and H_f_, and H_8_ and H_e_.

### Study on the directionally selective mechanism

These results were exciting but not beyond expectation, and could be tentatively explained as follows. In the threading process of **G1** (the bipyridinium salt), the electrostatic effect dominated the threading direction while the steric effect posed an opposite influence. As a result, a pair of orientationally isomeric rotaxanes in a ratio of 4 : 1 was obtained, and in the major product the pre-stoppered end was in proximity to the lower rim of **H**. However, when axle **G3** (the pyridinium salt) threaded into the macrocycle, the electrostatic effect and the steric effect acted in a cooperative way, generating a single compound in which the pre-stoppered end was in proximity to the upper rim of **H**. Moreover, to manifest the high selectivity in the threading direction of **G3** into **H** in an *in situ* manner, a variable-temperature ^1^H NMR experiment on the CDCl_3_/CD_3_CN solution of the pseudorotaxane based on **G3** and **H** (**G3** : **H** = 1.5 : 1) was conducted (Fig. S43–45, ESI†). From the spectra, we observed that when the temperature was brought down to approximately 233 K, the peaks of the free guest started to appear, indicating that the threading and dethreading had become a slow exchange process on the NMR timescale. Meanwhile, it was found that the pseudorotaxane still appeared as a single set of signals, which meant that at and below this temperature, the ^1^H NMR spectra clearly showed that the threading of **G3** into **H** occurred unidirectionally.¶
¶As the signals of the pseudorotaxane changed gradually with decreasing temperature without saltation of each chemical shift, we could safely deduce that the threading direction did not change during the cooling process.


DFT theoretical modeling was carried out in order to establish whether, for each axle, the orientational outcome is dictated by kinetic factors or by the different thermodynamic stability of the two isomers.† We calculated the relative Gibbs free energies of the two orientational isomers of pseudorotaxane **H**@**G1** (**PR1a** and **PR1b**), and found that the free energy of **PR1a** (corresponding to the orientation of **R1a**) was lower than that of its isomer **PR1b** by only 0.9 kcal mol^–1^. However, for the pseudorotaxanes based on **G3** and **H** (**PR3a** and **PR3b**), the calculations indicated that the relative Gibbs free energy of **PR3b** (corresponding to the orientation of rotaxane **R3b**) was lower than that of **PR3a** by a sizable 3.6 kcal mol^–1^. Thus, the DFT calculations verified the relative stabilities of the two pairs of isomers: both dominant isomers (**PR1a** and **PR3b**) were the relatively more stable ones, though **PR1a** only had a small advantage over **PR1b** energetically.

**Fig. 4:**
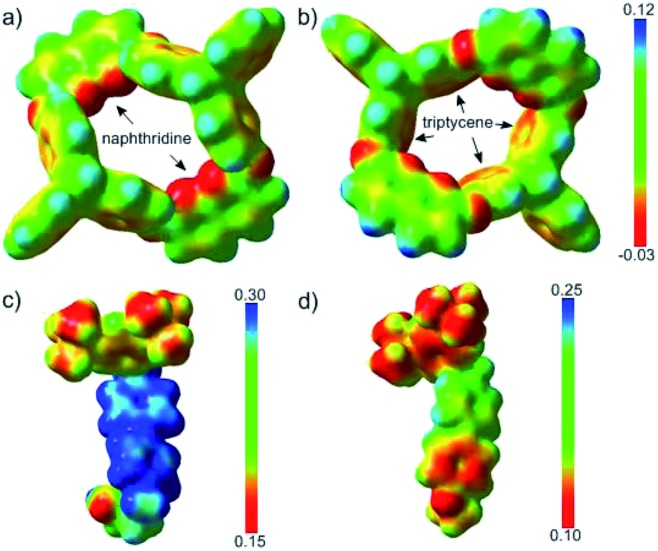
ESPs mapped onto electron density isosurfaces (*ρ* = 0.01) for **H** viewed from the lower rim (a) and upper rim (b), and for **G1** (c) and **G3** (d).

The ESPs of **H** in the absence of guest molecules were also calculated, as depicted in Fig. 4a and b. Two features were notable; one was the electron-rich character of the phenyl rings of the triptycene semi-cavity, and the other was the polarity of the naphthyridine moieties, characterized by the electron-rich N atoms and the electron-poor remaining part. The difference in the electron densities of guests **G1** and **G3** was visualized by comparing their ESPs (Fig. 4c and d). **G3** had a clearly higher electron density than **G1**, especially with respect to the unstoppered end (the phenyl ring). Therefore, the structures revealed the electrostatic repulsion of the electron-poor pyridinyl ring (the one further from the pre-stopper) in **G1** and the attraction of the electron-rich phenyl ring in **G3** exerted by the upper semi-cavity, and also the repulsion of the phenyl ring in **G3** by the lower semi-cavity, in the threading processes of the two guests.

From the above calculations, we concluded that the two isomers of **H**@**G1** had similar stability, but in forming **PR1b** from the less crowded upper rim, the barrier posed by the electrostatic effect might be higher than that in forming **PR1a**, while the subordinate factor, the steric effect, acted oppositely; as a result, two isomers in a ratio of 4 : 1 were obtained. However, with regard to **H**@**G3**, on the one hand **PR3b** was more stable than its isomer by 3.6 kcal mol^–1^, and on the other hand, threading from the upper rim was favorable in terms of both electrostatic and steric effects; thus, unidirectional threading was attained.

As we hypothesized that the directional selectivity in the **H**@**G1** system resulted mainly from the difference in energy barriers in the threading processes in each direction, increasing the temperature ought to narrow the gap between them. To verify this consideration, rotaxanes based on **G1** and **H** were synthesized at raised temperatures (313 K and 333 K), and the ratios of the isomers were determined *via*^1^H NMR analysis (Fig. S29–30, ESI†). In agreement with our prediction, the directional selectivity in the threading process of **G1** decreased with the increase in temperature (Table 1). However, in the same experiments on **G3** and **H**, the orientational isomer of **R3b** could not even be traced. The results further demonstrated that at ambient temperature the threading of **G1** was kinetically controlled.

**Table 1:** Ratios of **R1a** and **R1b** obtained at different reaction temperatures

Entry	Temperature (K)	Ratio of **R1a** and **R1b**^*a*^
1	298	4 : 1
2	313	1.7 : 1
3	333	1.2 : 1

^*a*^Determined by ^1^H NMR.

## Conclusions

In this work, we successfully demonstrated that the threading direction of (bi)pyridinium salts (**G1–G3**) into a triptycene-derived oxacalixarene (**H**) could be effectively controlled by tuning the electron densities of the guests, and the single crystal structures of (pseudo)rotaxanes exclusively manifested the orientation of the host and the guests. As the directional selectivity could be varied by tuning the roles played by steric and electrostatic effects, this work potentially provides a new approach towards the construction of oriented and well-aligned hierarchical assemblies. This is the first report on selective or unidirectional threading based on oxacalixarenes, and high directional selectivity was attained without either the use of special counteranions^5^,^13^ for the guest or further functionalization of the calixarene.^4^,^9^ The design of new guests and modification of the macrocycle by replacing the naphthyridine moieties with other electron-deficient moieties to obtain more precise control of directional selectivity from both sides, and the construction of high-order mechanically interlocked structures capable of performing unidirectional motion, are currently under investigation in our laboratory.

## Experimental

### General remarks

The synthesis of precursors of the rotaxanes is described in the ESI.† All the other reagents and solvents were bought from commercial suppliers and used as received.

### Synthesis of **R1a** and **R1b**

A mixture of **H** (62 mg, 0.075 mmol) and **G1** (53 mg, 0.075 mmol) in chloroform (10 mL) and acetonitrile (5 mL) was stirred at ambient temperature for 12 h. 3,5-Di-*tert*-butylbenzoic anhydride (135 mg, 0.3 mmol) and (*n*-Bu)_3_P (3 mg, 0.015 mmol) were then added to the mixture. The reaction mixture was stirred under argon for 24 h at ambient temperature. The solvent was removed under vacuum, and the residue was purified by column chromatography (CH_2_Cl_2_/acetone, 100 : 1 v/v) to give **R1a** and **R1b** as a mixture (73 mg) in 56% total yield. The macrocycle in the unreacted pseudorotaxanes could be recycled nearly quantitatively. The mixture was further separated carefully by preparative thin-layer chromatography to give pure **R1a** and **R1b**, both as yellow powders, for characterization.

#### **R1a**

Mp: 229–232 °C. ^1^H NMR (300 MHz, CDCl_3_): *δ* 9.03 (d, *J* = 6.3 Hz, 2H), 8.45 (d, *J* = 6.4 Hz, 2H), 8.33 (d, *J* = 6.3 Hz, 2H), 8.00 (d, *J* = 8.6 Hz, 4H), 7.97 (s, 2H), 7.71 (s, 1H), 7.57 (s, 2H), 7.51 (m, 3H), 7.20 (d, *J* = 6.7 Hz, 2H), 7.13 (d, *J* = 6.7 Hz, 2H), 7.04–7.05 (m, 4H), 6.92 (d, *J* = 8.6 Hz, 4H), 6.72–6.83 (m, 8H), 6.40 (d, *J* = 7.9 Hz, 4H), 5.79 (s, 2H), 5.24 (s, 4H), 4.25 (t, *J* = 6.8 Hz, 2H), 3.94 (t, *J* = 5.8 Hz, 2H), 1.93–2.02 (m, 2H), 1.36 (s, 18H), 1.29 (s, 18H). ^13^C NMR (75 MHz, CDCl_3_): *δ* 166.8, 164.1, 153.6, 153.3, 151.3, 151.2, 150.7, 149.1, 147.8, 146.7, 145.3, 145.1, 144.8, 144.3, 141.4, 140.3, 131.2, 128.5, 127.9, 127.4, 126.1, 125.3, 124.9, 124.4, 124.2, 123.7, 123.5, 117.2, 116.4, 116.0, 112.6, 60.4, 58.0, 57.0, 53.2, 51.4, 35.1, 34.9, 31.7, 31.3, 29.7. ESI-HRMS calcd for C_99_H_90_N_6_O_6_ [M – 2PF_6_^–^]^2+^ 729.8472; found 729.8485.

#### **R1b**

Mp: 223–225 °C. ^1^H NMR (300 MHz, CDCl_3_): *δ* 8.58 (d, *J* = 6.3 Hz, 2H), 8.20–8.22 (m, 6H), 8.06 (s, 2H), 7.82 (d, *J* = 6.3 Hz, 2H), 7.76 (s, 1H), 7.67 (d, *J* = 6.3 Hz, 2H), 7.55 (s, 1H), 7.21–7.30 (m, 4H), 7.14–7.16 (m, 4H), 7.12 (s, 2H), 7.02–7.05 (m, 8H), 6.88–6.91 (m, 4H), 6.67 (d, *J* = 7.9 Hz, 4H), 5.53 (s, 2H), 5.37 (s, 2H), 5.32 (s, 2H), 4.28 (t, *J* = 6.2 Hz, 2H), 4.03 (t, *J* = 6.3 Hz, 2H), 1.99–2.02 (m, 2H), 1.38 (s, 18H), 1.29 (s, 18H). ^13^C NMR (125 MHz, CDCl_3_): *δ* 167.4, 164.0, 153.1, 151.7, 151.2, 148.7, 148.3, 147.2, 145.0, 144.8, 144.6, 144.3, 141.6, 141.2, 130.1, 129.7, 129.5, 129.0, 128.1, 125.4, 125.3, 125.0, 124.5, 124.1, 124.0, 123.7, 123.4, 117.8, 116.6, 115.9, 113.4, 60.6, 58.6, 54.8, 52.9, 52.2, 35.1, 35.0, 31.4, 31.3, 29.7. ESI-HRMS calcd for C_99_H_90_N_6_O_6_ [M – 2PF_6_^–^]^2+^ 729.8472; found 729.8454.

### Synthesis of **R2a** and **R2b**

A mixture of **H** (62 mg, 0.075 mmol) and **G2** (56 mg, 0.075 mmol) in chloroform (10 mL) and acetonitrile (5 mL) was stirred at ambient temperature for 12 h. 3,5-Di-*tert*-butylbenzoic anhydride (135 mg, 0.3 mmol) and (*n*-Bu)_3_P (3 mg, 0.015 mmol) were then added to the mixture. The reaction mixture was stirred under argon for 24 h at ambient temperature. The solvent was removed under vacuum, and the residue was purified by column chromatography (CH_2_Cl_2_/acetone, 100 : 1 v/v) to give **R2a** and **R2b** as a mixture (71 mg) in 53% total yield. The macrocycle in the unreacted pseudorotaxanes could be recycled nearly quantitatively. Then, the mixture was carefully separated by preparative thin-layer chromatography to give a fraction of pure **R2a** as a yellow powder, but it turned out to be difficult to obtain pure **R2b** for further characterization.

#### **R2a**

Mp: 208–210 °C. ^1^H NMR (600 MHz, CDCl_3_): *δ* 8.96 (brs, 2H), 8.39 (brs, 2H), 8.27 (brs, 2H), 8.00 (d, *J* = 7.8 Hz, 4H), 7.88 (s, 2H), 7.63 (s, 1H), 7.57–7.61 (m, 4H), 7.52 (s, 1H), 7.18 (d, *J* = 7.2 Hz, 2H), 7.09 (d, *J* = 7.2 Hz, 2H), 7.01 (brs, 4H), 6.90 (d, *J* = 7.8 Hz, 4H), 6.70–6.79 (m, 8H), 6.44 (d, *J* = 7.5 Hz, 4H), 5.77 (s, 2H), 5.25 (s, 2H), 5.22 (s, 2H), 4.09 (t, *J* = 7.8 Hz, 2H), 4.01 (brs, 2H), 1.48–1.50 (m, 2H), 1.35–1.38 (m, 2H), 1.31 (s, 18H), 1.27 (s, 18H), 1.07–1.10 (m, 2H), 0.84–0.87 (m, 2H). ^13^C NMR (75 MHz, CDCl_3_): *δ* 167.3, 164.0, 153.5, 153.2, 151.2, 150.5, 148.9, 147.5, 146.7, 145.2, 145.0, 144.7, 144.4, 141.5, 140.4, 131.5, 129.6, 127.5, 127.2, 126.1, 125.3, 124.9, 124.8, 124.2, 123.7, 123.56, 123.52, 117.2, 116.4, 116.2, 112.5, 64.3, 60.8, 53.1, 51.5, 35.1, 34.9, 31.38, 31.35, 31.1, 29.7, 28.2, 25.2, 25.0. ESI-HRMS calcd for C_102_H_96_N_6_O_6_ [M – 2PF_6_^–^]^2+^ 750.8707; found 750.8716.

### Synthesis of **R3b**

A mixture of **H** (62 mg, 0.075 mmol) and **G3** (43 mg, 0.075 mmol) in a solution of chloroform (10 mL) and acetonitrile (5 mL) was stirred at ambient temperature for 12 h. 3,5-Di-*tert*-butylbenzoic anhydride (135 mg, 0.3 mmol) and (*n*-Bu)_3_P (3 mg, 0.015 mmol) were then added to the mixture. The reaction mixture was stirred under argon for 24 h at ambient temperature. The solvent was removed under vacuum, and the residue was purified by column chromatography (CH_2_Cl_2_/acetone, 100 : 1 v/v) to give **R3b** (63 mg, 52% yield) as a white powder. The macrocycle in the unreacted pseudorotaxanes could be recycled nearly quantitatively. Mp: 211–213 °C. ^1^H NMR (400 MHz, CDCl_3_): *δ* 8.08 (d, *J* = 8.6 Hz, 4H), 8.03 (d, *J* = 8.5 Hz, 2H), 8.00 (s, 2H), 7.79 (d, *J* = 6.2 Hz, 2H), 7.66 (s, 1H), 7.53 (d, *J* = 6.2 Hz, 2H), 7.40 (d, *J* = 8.5 Hz, 2H), 7.23 (s, 1H), 7.17–7.23 (m, 4H), 7.02 (d, *J* = 8.6 Hz, 4H), 6.98 (brs, 4H), 6.87–6.89 (m, 4H), 6.79–6.85 (m, 8H), 6.55 (s, 2H), 5.15 (s, 2H), 4.95 (s, 2H), 4.86 (brs, 4H), 4.61 (t, *J* = 4.2 Hz, 2H), 1.35 (s, 18H), 1.08 (s, 18H). ^13^C NMR (75 MHz, CDCl_3_): *δ* 167.3, 164.1, 162.1, 153.8, 153.0, 151.9, 151.2, 150.1, 146.2, 145.3, 144.6, 141.9, 141.0, 140.0, 130.5, 129.9, 129.3, 127.4, 126.9, 125.3, 125.0, 124.0, 123.7, 123.55, 123.50, 123.4, 117.1, 117.0, 115.7, 112.3, 66.6, 62.7, 53.5, 52.3, 35.0, 34.6, 31.4, 31.1, 29.3. ESI-HRMS calcd for C_99_H_88_N_5_O_7_ [M – PF_6_^–^]^+^ 1458.6678; found 1458.6679.

## Supplementary Material

Supplementary information

Crystal structure data
