# Supplementary material for: Guest-dependent directional complexation based on triptycene derived oxacalixarene: formation of oriented rotaxanes
Source: Chem Sci. 2015 Oct 8;7(1):469–74. doi: 10.1039/c5sc03511b (PMC5964692; doi:10.1039/c5sc03511b)
Supplement: Supplementary file 1 [file SC-007-C5SC03511B-s001.pdf]

Electronic Supplementary Material (ESI) for Chemical Science.
This journal is © The Royal Society of Chemistry 2015

Electronic Supplementary Information

Guest-dependent directional complexation based on triptycene derived oxacalixarene: formation of oriented rotaxanes

Han-Xiao Wang,[a,c] Zheng Meng,[a,c] Jun-Feng Xiang,[a] Yu-Xiang Xia,[a] Yihua Sun,[b] Shu-Zhen Hu,[a] Hui Chen,*[b] Jiannian Yao,[b] and Chuan-Feng Chen*[a]

[a]*Beijing National Laboratory for Molecular Sciences, CAS Key Laboratory of Molecular Recognition and Function, Institute of Chemistry, Chinese Academy of Sciences, Beijing 100190, China.* [b]*CAS Key Laboratory of Photochemistry, Institute of Chemistry, Chinese Academy of Sciences, Beijing 100190, China.* [c]*University of Chinese Academy of Sciences, Beijing 100049, China.*

E-mail: cchen@iccas.ac.cn

Contents

1. **Synthesis and characterization data of new compounds**
2. **Copies of ^{1}H NMR and ^{13}C NMR spectra of new compounds**
3. **Copies of 2D NMR spectra of rotaxanes**
4. **Copies of partial ^{1}H NMR spectra showing the ratios of isomers of R1a-b and R2a-b synthesized under different temperatures**
5. **^{1}H NMR spectra of complexation between host H and guests G1-G3**
6. **Determination of the stoichiometries and association constants**
7. **Variable-temperature ^{1}H NMR spectra of H@G3 and G3**
8. **Crystal data**
9. **Computational methods**
10. **Cartesian coordiates of computed structures**
11. **References**

1. Synthesis and characterization data of new compounds

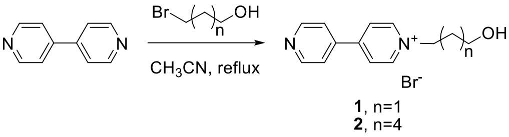

General procedure for preparation of 1 and 2. A mixture of 4,4′-bipyridine (156 mg, 1 mmol) and 3-bromopropanol (552 mg, or 6-bromohexanol, 720 mg, 4 mmol) was refluxed in acetonitrile (50 mL) for 24 h. After cooling to room temperature, the mixture was filtrated, and the residue was washed with ethyl acetate to afford the product (273 mg for **1**, 93% yield; and 306 mg for **2**, 91% yield) as white powder.

1: Mp: 172-174 ℃. ^{1}H NMR (300 MHz, D_2O): δ 9.05 (d, *J* = 6.1 Hz, 2H), 8.74 (d, *J* = 4.1 Hz, 2H), 8.43 (d, *J* = 6.1 Hz, 2H), 7.91 (d, *J* = 4.1 Hz, 2H), 4.85 (t, *J*= 7.4 Hz, 2H), 3.76-3.79 (m, 2H), 2.32-2.41 (m, 2H). ^{13}C NMR (75 MHz, D_2O): δ 153.5, 150.0, 145.0, 142.3, 126.0, 122.5, 58.9, 57.9, 32.7. ESI-HRMS calcd for $C_{13}H_{15}N_2O$ $[M\text{-}Br^-]^+$ 215.1179; found 215.1178.

2: Mp: 162-163 ℃. ^{1}H NMR (300 MHz, D_2O): δ 9.00 (d, *J* = 6.7 Hz, 2H), 8.77 (d, *J* = 6.3 Hz, 2H), 8.42 (d, *J* = 6.7 Hz, 2H), 7.92 (d, *J* = 6.3 Hz, 2H), 4.70 (t, *J* = 7.3 Hz, 2H), 3.61 (t, *J* = 6.4 Hz, 2H), 2.08-2.13 (m, 2H), 1.55-1.60 (m, 2H), 1.41-1.46 (m, 4H). ^{13}C NMR (75 MHz, D_2O): δ 153.7, 149.9, 144.7, 142.7, 126.0, 122.5, 61.6, 61.5, 31.0, 30.4, 25.0, 24.5. ESI-HRMS calcd for $C_{16}H_{21}N_2O$ $[M\text{-}Br^-]^+$ 257.1648; found 257.1646.

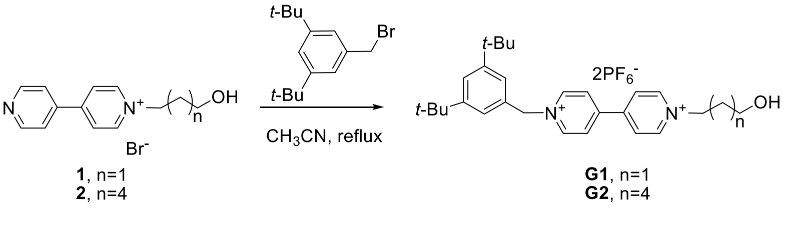

General procedure for preparation of G1 and G2. A mixture of **1** (147 mg, or **2**, 168 mg, 0.5mmol) and 1-(bromomethyl)-3,5-di-*tert*-butylbenzene[1] (212 mg, 0.75 mmol) was refluxed in acetonitrile (25 mL) for 72 h. After cooling to room temperature, the reaction mixture was filtrated, and the residue was washed with ethyl acetate. The crude product was dispersed in acetone, and then treated with excess saturated aqueous solution of NH_4PF_6. The solution was stirred at ambient temperature until clear. The acetone was removed under vacuum, and the residue was

then washed with water to afford **G1** (312 mg, 88% yield) and **G2** (323 mg, 86% yield) as white powder.

G1: Mp: 214-216 ℃. ^{1}H NMR (300 MHz, CD_3CN): δ 8.99 (d, J = 6.4 Hz, 2H, -C_5H_4N-), 8.93 (d, J = 6.4 Hz, 2H, -C_5H_4N-), 8.36-8.37 (m, 4H, -C_5H_4N-), 7.59 (s, 1H, Ar*H*), 7.41 (s, 2H, Ar*H*), 5.77 (s, 2H, -C_5H_4N-CH_2-Ar), 4.75 (t, J = 6.9 Hz, 2H, -C_5H_4N-CH_2-CH_2), 3.57-3.62 (m, 2H, -OCH_2), 2.90 (t, J = 4.9 Hz, 1H, -*OH*), 2.15-2.24 (m, 2H, -CH_2C$H_2$$CH_2$-), 1.33 (s, 18H, Ar-*t-Bu*). ^{13}C NMR (75 MHz, CD_3CN): δ 153.6, 151.2, 150.8, 146.9, 146.4, 132.9, 128.4, 128.0, 125.3, 124.8, 66.4, 60.8, 58.4, 35.7, 33.8, 31.5. ESI-HRMS calcd for $C_{28}H_{38}F_6N_2OP$ [M-PF_6^-]$^+$ 563.2626; found 563.2612.

G2: Mp: 192-193 ℃. ^{1}H NMR (300 MHz, CD_3CN): δ 9.00 (d, J = 6.4 Hz, 2H, -C_5H_4N-), 8.89 (d, J = 6.4 Hz, 2H, -C_5H_4N-), 8.37-8.39 (m, 4H, -C_5H_4N-), 7.59 (s, 1H, Ar*H*), 7.42 (s, 2H, Ar*H*), 5.77 (s, 2H, -C_5H_4N-CH_2-Ar), 4.61 (t, J = 7.5 Hz, 2H, -C_5H_4N-CH_2-CH_2), 3.49 (t, J = 6.1 Hz, 2H, -OCH_2-), 2.48 (brs, 1H, -*OH*), 2.00-2.09 (m, 2H, -CH_2C$H_2$$CH_2$-), 1.45-1.54 (m, 2H, -C$H_2CH_2CH_2$-), 1.38-1.42 (m, 4H, -C$H_2CH_2CH_2$-), 1.33 (s, 18H, Ar-*t*-Bu). ^{13}C NMR (75 MHz, CD_3CN): δ 153.6, 151.9, 151.3, 151.1, 150.9, 146.4, 132.9, 128.4, 128.2, 125.3, 124.9, 66.4, 63.1, 62.3, 35.8, 33.1, 31.9, 31.5, 26.4, 25.9. ESI-HRMS calcd for $C_{31}H_{44}F_6N_2OP$ [M-PF_6^-]$^+$ 605.3095; found 605.3075.

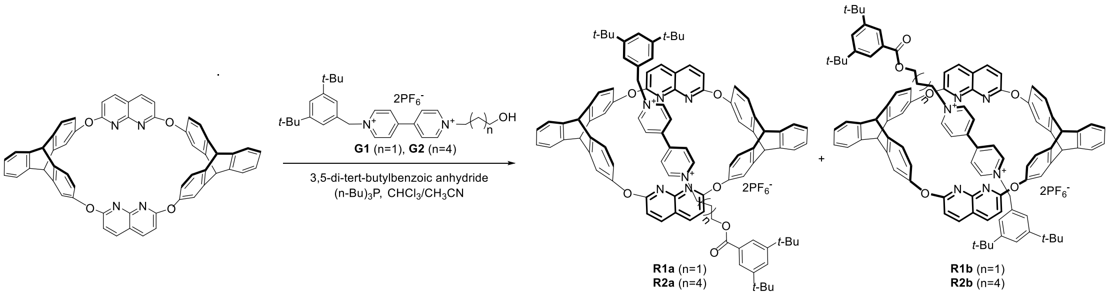

Synthesis of R1a and R1b. A mixture of **H** (62 mg, 0.075 mmol) and **G1** (53 mg, 0.075 mmol) in chloroform (10 mL) and acetonitrile (5 mL) was stirred at ambient temperature for 12h. To the mixture was then added 3,5-di-*tert*-butylbenzoic anhydride (135 mg, 0.3 mmol) and (n-Bu)$_3$P (3 mg, 0.015 mmol). The reaction mixture was stirred under argon for 24 h at ambient temperature. The solvent was removed under vacuum, and the residue was purified by column chromatography (dichloromethane/acetone, 100:1 *v/v*) to give **R1a** and **R1b** as a mixture (73 mg) in total 56% yield. The macrocycle in unreacted pseudorotaxanes could be recycled nearly quantitatively. The

mixture was further separated carefully by preparative thin-layer chromatography to give pure **R1a** and **R1b** both as yellow powder for characterization.

R1a: Mp: 229-232 ℃. ^{1}H NMR (300 MHz, $CDCl_3$): δ 9.03 (d, J = 6.3 Hz, 2H, -C_5H_4N-), 8.45 (d, J = 6.4 Hz, 2H, -C_5H_4N-), 8.33 (d, J = 6.3 Hz, 2H, -C_5H_4N-), 8.00 (d, J = 8.6 Hz, 4H, naphthyridine Ar*H*), 7.97 (s, 2H, stopper Ar*H*), 7.71 (s, 1H, stopper Ar*H*), 7.57 (s, 2H, stopper Ar*H*), 7.51 (m, 3H, 2H for -C_5H_4N-, 1H for stopper Ar*H*), 7.20 (d, J = 6.7 Hz, 2H, triptycene Ar*H*), 7.13 (d, J = 6.7 Hz, 2H, triptycene Ar*H*), 7.04-7.05 (m, 4H, triptycene Ar*H*), 6.92 (d, J = 8.6 Hz, 4H, naphthyridine Ar*H*), 6.72-6.83 (m, 8H, triptycene Ar*H*), 6.40 (d, J = 7.9 Hz, 4H, triptycene Ar*H*), 5.79 (s, 2H, -C_5H_5N-CH_2-Ar), 5.24 (s, 4H, Ar_3C*H*), 4.25 (t, J =6.8 Hz, 2H, -C_5H_5N-CH_2-CH_2), 3.94 (t, J =5.8 Hz, 2H, -OCH_2), 1.93-2.02 (m, 2H, -CH_2C$H_2$$CH_2$-), 1.36 (s, 18H, Ar-*t-Bu*), 1.29 (s, 18H, Ar-*t-Bu*). ^{13}C NMR (75 MHz, $CDCl_3$): δ 166.8, 164.1, 153.6, 153.3, 151.3, 151.2, 150.7, 149.1, 147.8, 146.7, 145.3, 145.1, 144.8, 144.3, 141.4, 140.3, 131.2, 128.5, 127.9, 127.4, 126.1, 125.3, 124.9, 124.4, 124.2, 123.7, 123.5, 117.2, 116.4, 116.0, 112.6, 60.4, 58.0, 57.0, 53.2, 51.4, 35.1, 34.9, 31.7, 31.3, 29.7. ESI-HRMS calcd for $C_{99}H_{90}N_6O_6$ $[M-2PF_6^-]^{2+}$ 729.8472; found 729.8485.

R1b: Mp: 223-225 ℃. ^{1}H NMR (300 MHz, $CDCl_3$): δ 8.58 (d, J = 6.3 Hz, 2H, -C_5H_4N-), 8.20-8.22 (m, 6H, 4H for naphthyridine Ar*H* and 2H for-C_5H_4N-), 8.06 (s, 2H, stopper Ar*H*), 7.82 (d, J = 6.3 Hz, 2H, -C_5H_4N-), 7.76 (s, 1H, stopper Ar*H*), 7.67 (d, J = 6.3 Hz, 2H, -C_5H_4N-), 7.55 (s, 1H, stopper Ar*H*), 7.21-7.30 (m, 4H, triptycene Ar*H*), 7.14-7.16 (m, 4H, triptycene Ar*H*), 7.12 (s, 2H, stopper Ar*H*), 7.02-7.05 (m, 8H, 4H for naphthyridine Ar*H* and 4H for triptycene Ar*H*), 6.88-6.91 (m, 4H, triptycene Ar*H*), 6.67 (d, J = 7.9 Hz, 4H, triptycene Ar*H*), 5.53 (s, 2H, -C_5H_5N-CH_2-Ar), 5.37 (s, 2H, Ar_3C*H*), 5.32 (s, 2H, Ar_3C*H*), 4.28 (t, J = 6.2 Hz, 2H, -C_5H_5N-CH_2-CH_2), 4.03 (t, J = 6.3 Hz, 2H, -OCH_2), 1.99-2.02 (m, 2H, -CH_2C$H_2$$CH_2$-), 1.38 (s, 18H, Ar-*t-Bu*), 1.29 (s, 18H, Ar-*t-Bu*). ^{13}C NMR (125 MHz, $CDCl_3$): δ 167.4, 164.0, 153.1, 151.7, 151.2, 148.7, 148.3, 147.2, 145.0, 144.8, 144.6, 144.3, 141.6, 141.2, 130.1, 129.7, 129.5, 129.0, 128.1, 125.4, 125.3, 125.0, 124.5, 124.1, 124.0, 123.7, 123.4, 117.8, 116.6, 115.9, 113.4, 60.6, 58.6, 54.8, 52.9, 52.2, 35.1, 35.0, 31.4, 31.3, 29.7. ESI-HRMS calcd for $C_{99}H_{90}N_6O_6$ $[M-2PF_6^-]^{2+}$ 729.8472; found 729.8454.

Synthesis of R2a and R2b. A mixture of **H** (62 mg, 0.075 mmol) and **G2** (56 mg, 0.075 mmol) in chloroform (10 mL) and acetonitrile (5 mL) was stirred at ambient temperature for 12h. To the mixture was then added 3,5-di-*tert*-butylbenzoic anhydride (135 mg, 0.3 mmol) and (n-Bu)$_3$P (3 mg, 0.015 mmol). The reaction mixture was stirred under argon for 24 h at ambient temperature. The solvent was removed under vacuum, and the residue was purified by column chromatography (dichloromethane/acetone, 100:1 *v/v*) to give **R2a** and **R2b** as a mixture (71 mg) in total 53% yield. The macrocycle in unreacted pseudorotaxanes could be recycled nearly quantitatively. Then, the mixture was carefully separated by preparative thin-layer chromatography to give a fraction of pure **R2a** as yellow powder, but it turned out to be difficult to obtain pure **R2b** for further characterization.

R2a: Mp: 208-210 ℃. ^{1}H NMR (600 MHz, $CDCl_3$): δ 8.96 (brs, 2H, -C_5H_4N-), 8.39 (brs, 2H, -C_5H_4N-), 8.27 (brs, 2H, -C_5H_4N-), 8.00 (d, J = 7.8 Hz, 4H, naphthyridine Ar*H*), 7.88 (s, 2H, stopper Ar*H*), 7.63 (s, 1H, stopper Ar*H*), 7.57-7.61 (m, 4H, 2H for stopper Ar*H* and 2H for -C_5H_4N-), 7.52 (s, 1H, stopper Ar*H*), 7.18 (d, J = 7.2 Hz, 2H, triptycene Ar*H*), 7.09 (d, J = 7.2 Hz, 2H, triptycene Ar*H*), 7.01 (brs, 4H, triptycene Ar*H*), 6.90 (d, J = 7.8 Hz, 4H, naphthyridine Ar*H*), 6.70-6.79 (m, 8H, triptycene Ar*H*), 6.44 (d, J = 7.5 Hz, 4H, triptycene Ar*H*), 5.77 (s, 2H, -C_5H_5N-CH_2-Ar), 5.25 (s, 2H, Ar_3C*H*), 5.22 (s, 2H, Ar_3C*H*), 4.09 (t, J = 7.8 Hz, 2H, -C_5H_5N-CH_2-CH_2), 4.01 (brs, 2H, -OCH_2), 1.48-1.50 (m, 2H, -CH_2C$H_2$$CH_2$-), 1.35-1.38 (m, 2H, -$CH_2CH_2$$CH_2$-), 1.31 (s, 18H, Ar-*t*-*Bu*), 1.27 (s, 18H, Ar-*t*-*Bu*), 1.07-1.10 (m, 2H, -CH_2C$H_2$$CH_2$-), 0.84-0.87 (m, 2H, -$CH_2CH_2$$CH_2$-). ^{13}C NMR (75 MHz, $CDCl_3$): δ 167.3, 164.0, 153.5, 153.2, 151.2, 150.5, 148.9, 147.5, 146.7, 145.2, 145.0, 144.7, 144.4, 141.5, 140.4, 131.5, 129.6, 127.5, 127.2, 126.1, 125.3, 124.9, 124.8, 124.2, 123.7, 123.5(6), 123.5(2), 117.2, 116.4, 116.2, 112.5, 64.3, 60.8, 53.1, 51.5, 35.1, 34.9, 31.3(8), 31.3(5), 31.1, 29.7, 28.2, 25.2, 25.0. ESI-HRMS calcd for $C_{102}H_{96}N_6O_6$ [M-$2PF_6^-$]$^{2+}$ 750.8707; found 750.8716.

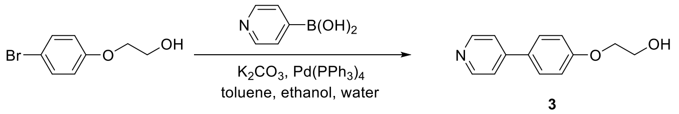

3

Synthesis of 3. To a mixture of 2-(4-bromophenoxy)ethanol[2] (1.08 g, 5 mmol), pyridine-4-boronic acid (676 mg, 5.5 mmol) and K_2CO_3 (2.07g, 15 mmol) in toluene (20 mL), ethanol (20

mL), and degassed water (10 mL) was added $Pd(PPh_3)_4$ (289 mg, 5.0 mol%). The mixture was then refluxed for 24 h. After the reaction mixture was cooled to room temperature, 50 mL dichloromethane and 50 mL water was added. The organic phase was separated, and the solvent was removed under vacuum. The residue was purified by column chromatography (petroleum/ethyl acetate, 2:1 *v/v*) to afford **3** (763 mg, 71% yield) as yellow powder. Mp: 181-182 ℃. ^{1}H NMR (300 MHz, $CDCl_3$): δ 8.62 (d, J = 6.0 Hz, 2H). 7.60 (d, J = 8.7 Hz, 2H), 7.47 (d, J = 6.0 Hz, 2H), 7.03 (d, J = 8.7 Hz, 2H), 4.14-4.17 (m, 2H), 3.99-4.02 (m, 2H), 2.23 (brs, 1H). ^{13}C NMR (75 MHz, $CDCl_3$): δ 159.7, 150.1, 147.8, 130.7, 128.2, 121.1, 115.2, 69.4, 61.3. ESI-HRMS calcd for $C_{13}H_{14}NO_2[M+H]^+$ 216.1024; found 216.1020.

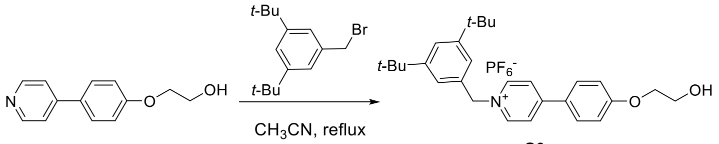

G3

Synthesis of G3. A mixture of **3** (215 mg, 1 mmol) and 1-(bromomethyl)-3,5-di-*tert*-butylbenzene (423 mg, 1.5 mmol) was refluxed in acetonitrile (50 mL) for 24 h. After cooling to room temperature, the solvent was removed under vacuum, the residue was washed with ethyl acetate, dispersed in acetone, and then treated with excess saturated aqueous solution of NH_4PF_6. The solution was stirred at ambient temperature until clear. The acetone was removed under vacuum, and the reaction mixture was then filtrated. The residue was washed with water to afford **G3** (479 mg, 85% yield) as white powder. Mp: 231-233 ℃. ^{1}H NMR (300 MHz, CD_3CN): δ 8.65 (d, J = 7.0 Hz, 2H, -C_5H_4N-), 8.18 (d, J = 7.0 Hz, 2H, -C_5H_4N-), 7.92 (d, J = 9.0 Hz, 2H, Ar*H*), 7.56 (s, 2H, Ar*H*), 7.36 (s, 2H, Ar*H*), 7.16 (d, J = 9.0 Hz, 2H, Ar*H*), 5.58 (s, 2H, -C_5H_4N-CH_2-Ar), 4.13-4.16 (m, 2H, -C$H_2$$CH_2$-), 3.82-3.87 (m, 2H, -$CH_2CH_2$-), 3.01 (brs, 1H, -*OH*), 1.32 (s, 18H, Ar-*t-Bu*). ^{13}C NMR (75 MHz, CD_3CN): δ 164.0, 156.9, 153.4, 144.8, 133.7, 131.1, 126.6, 125.0, 124.9, 124.5, 116.8, 71.1, 65.0, 61.2, 35.7, 31.5. ESI-HRMS calcd for $C_{28}H_{36}NO_2$ $[M-PF_6^-]^+$ 418.2741; found 418.2738.

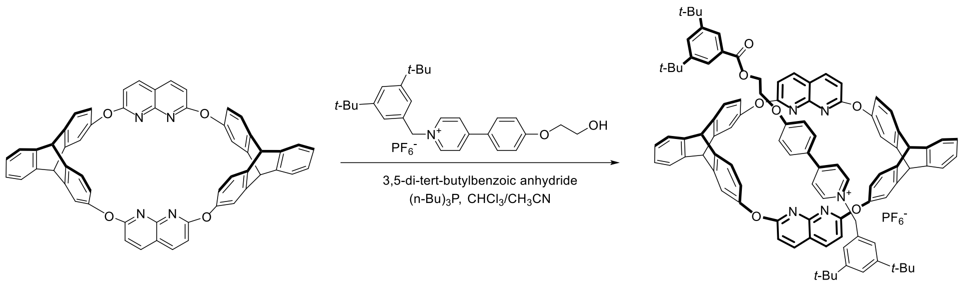

R3b

Synthesis of R3b. A mixture of **H** (62 mg, 0.075 mmol) and **G3** (43 mg, 0.075 mmol) in a solution of chloroform (10 mL) and acetonitrile (5 mL) was stirred at ambient temperature for 12h. Then, to the mixture was added 3,5-di-*tert*-butylbenzoic anhydride (135 mg, 0.3 mmol) and (*n*-Bu)$_3$P (3 mg, 0.015 mmol). The reaction mixture was stirred under argon for 24 h at ambient temperature. The solvent was removed under vacuum. The residue was purified by column chromatography (dichloromethane/acetone, 100:1 *v/v*) to give **R3b** (63 mg, 52% yield) as white powder. The macrocycle in unreacted pseudorotaxanes could be recycled nearly quantitatively. Mp: 211-213 ℃. ^{1}H NMR (400 MHz, CDCl$_3$): δ 8.08 (d, J = 8.6 Hz, 4H, naphthyridine Ar*H*), 8.03 (d, J = 8.5 Hz, 2H, -Ar*H*-C$_5$H$_4$N-), 8.00 (s, 2H, stopper Ar*H*), 7.79 (d, J = 6.2 Hz, 2H, -C$_5$*H*$_4$N-), 7.66 (s, 1H, stopper Ar*H*), 7.53 (d, J = 6.2 Hz, 2H, -C$_5$*H*$_4$N-), 7.40 (d, J = 8.5 Hz, 2H, -Ar*H*-C$_5$H$_4$N-), 7.23 (s, 1H, stopper Ar*H*), 7.17-7.23 (m, 4H, triptycene Ar*H*), 7.02 (d, J = 8.6 Hz, 4H, naphthyridine Ar*H*), 6.98 (brs, 4H, triptycene Ar*H*), 6.87-6.89 (m, 4H, triptycene Ar*H*), 6.79-6.85 (m, 8H, triptycene Ar*H*), 6.55 (s, 2H, stopper Ar*H*), 5.15 (s, 2H, Ar$_3$C*H*), 4.95 (s, 2H, Ar$_3$C*H*), 4.86 (brs, 4H, 2H for -OC*H*$_2$, 2H for C$_5$H$_5$N-C*H*$_2$-Ar), 4.61 (t, J = 4.2 Hz, 2H, -OC*H*$_2$), 1.35 (s, 18H, Ar-*t-Bu*), 1.08 (s, 18H, Ar-*t-Bu*). ^{13}C NMR (75 MHz, CDCl$_3$): δ 167.3, 164.1, 162.1, 153.8, 153.0, 151.9, 151.2, 150.1, 146.2, 145.3, 144.6, 141.9, 141.0, 140.0, 130.5, 129.9, 129.3, 127.4, 126.9, 125.3, 125.0, 124.0, 123.7, 123.5(5), 123.5(0), 123.4, 117.1, 117.0, 115.7, 112.3, 66.6, 62.7, 53.5, 52.3, 35.0, 34.6, 31.4, 31.1, 29.3. ESI-HRMS calcd for $C_{99}H_{88}N_5O_7$ [M-PF$_6^-$]$^+$ 1458.6678; found 1458.6679.

2. Copies of ^{1}H NMR and ^{13}C NMR spectra of new compounds[3]

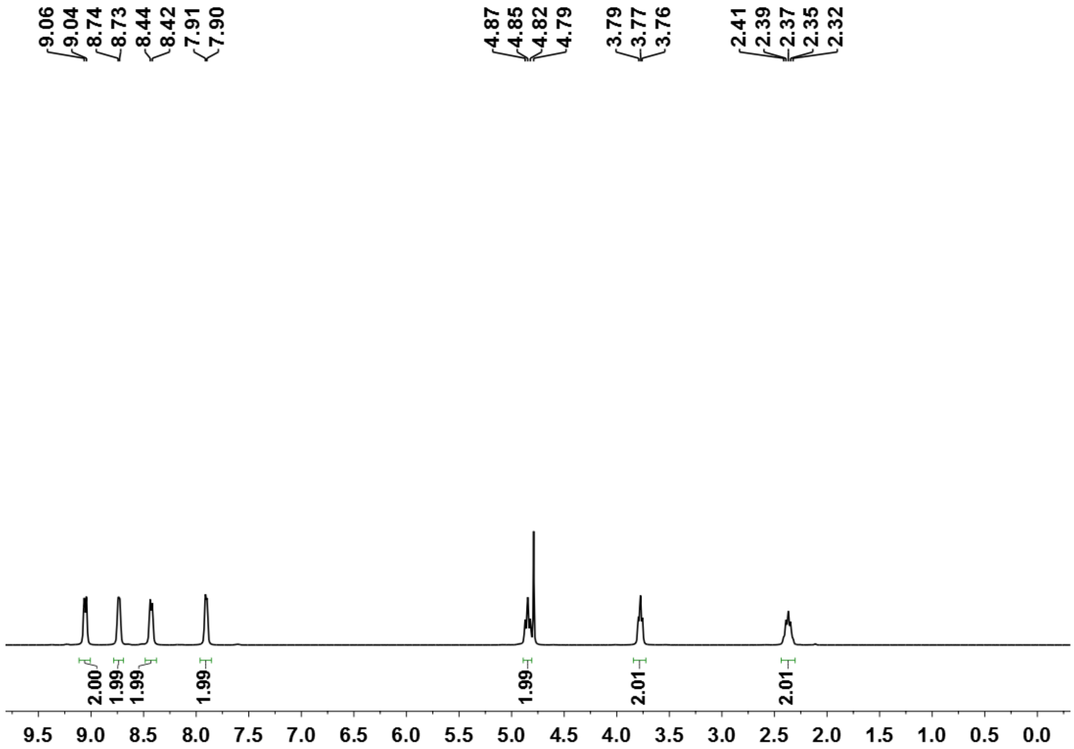

Fig. S1 ^{1}H NMR spectrum (300 MHz, D_2O) of **1**.

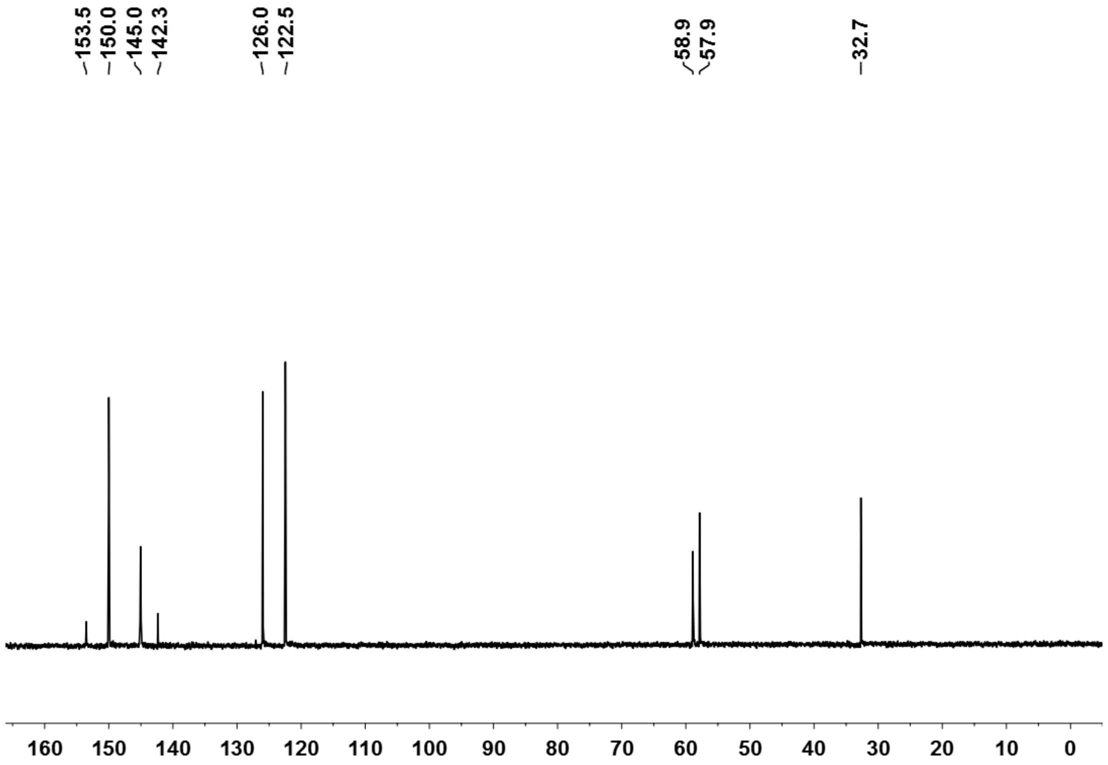

Fig. S2 ^{13}C NMR spectrum (75 MHz, D_2O) of **1**.

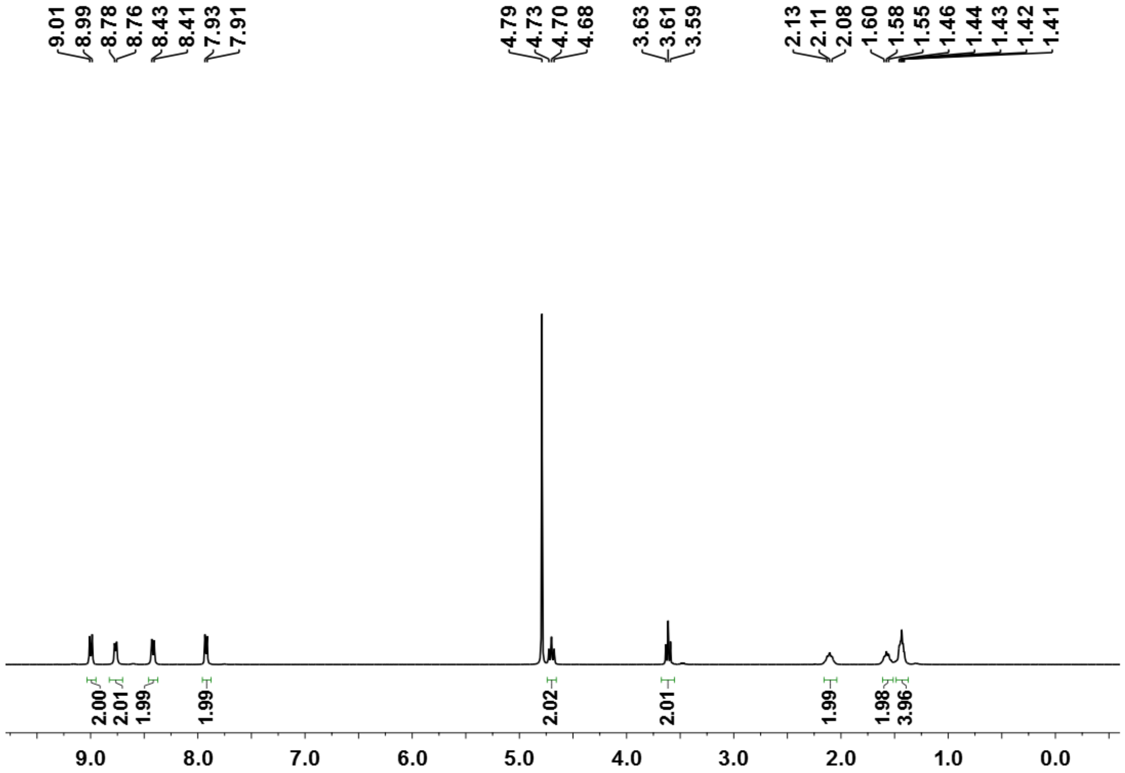

Fig. S3 ^{1}H NMR spectrum (300 MHz, D_2O) of **2**.

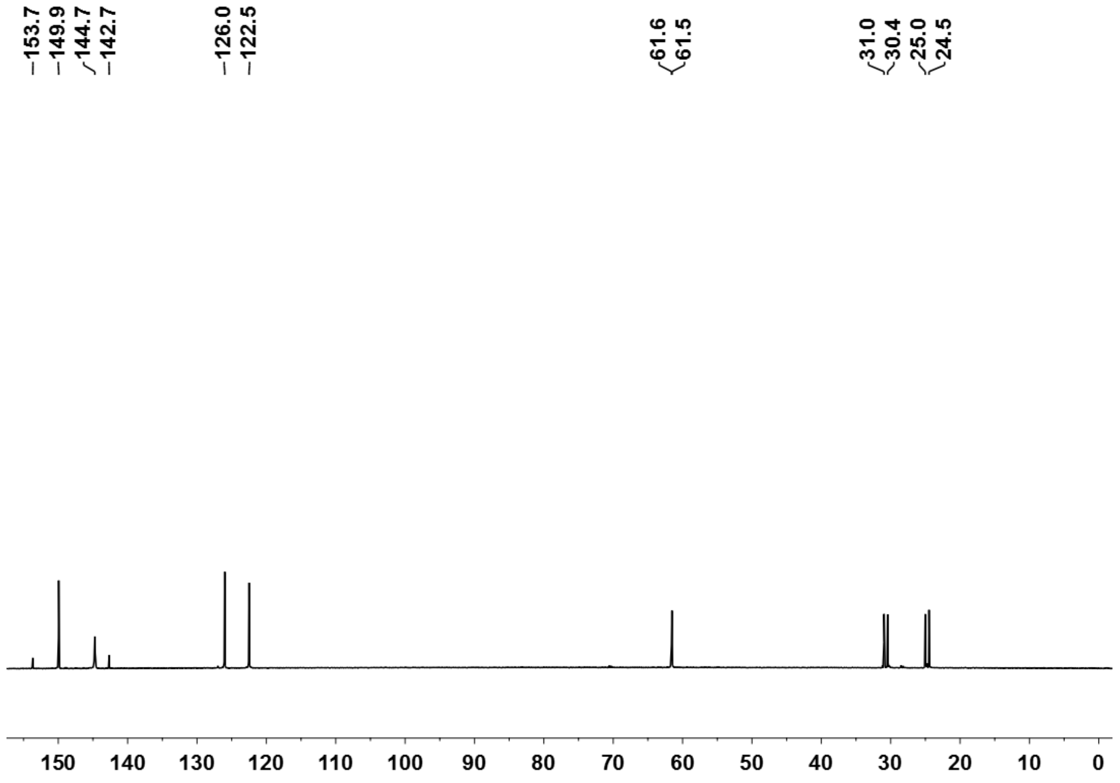

Fig. S4 ^{13}C NMR spectrum (75 MHz, D_2O) of **2**.

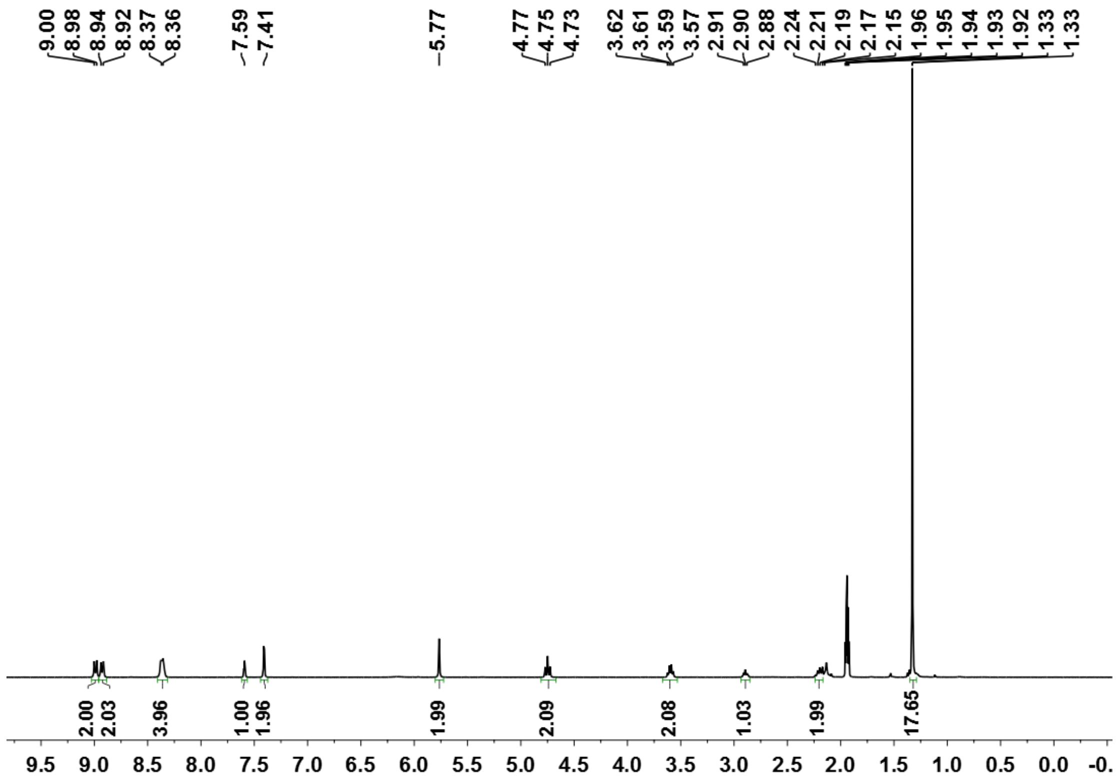

Fig. S5 ^{1}H NMR spectrum (300 MHz, CD_3CN) of **G1**.

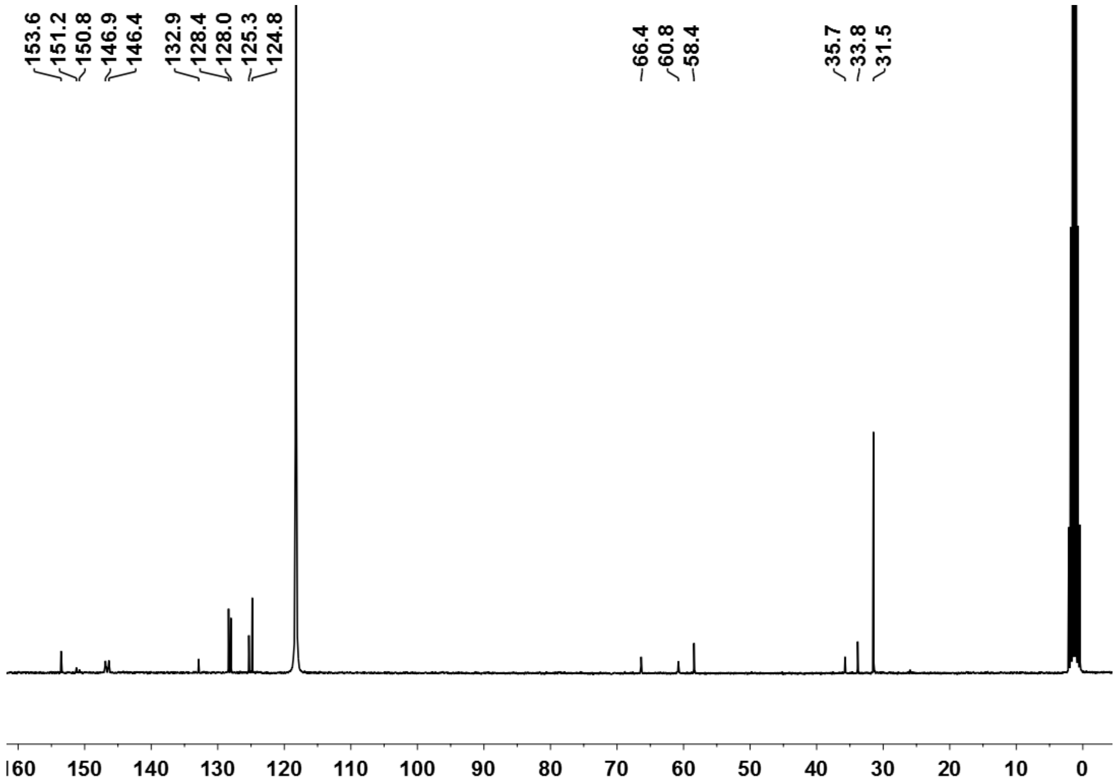

Fig. S6 ^{13}C NMR spectrum (75 MHz, CD_3CN) of **G1**.

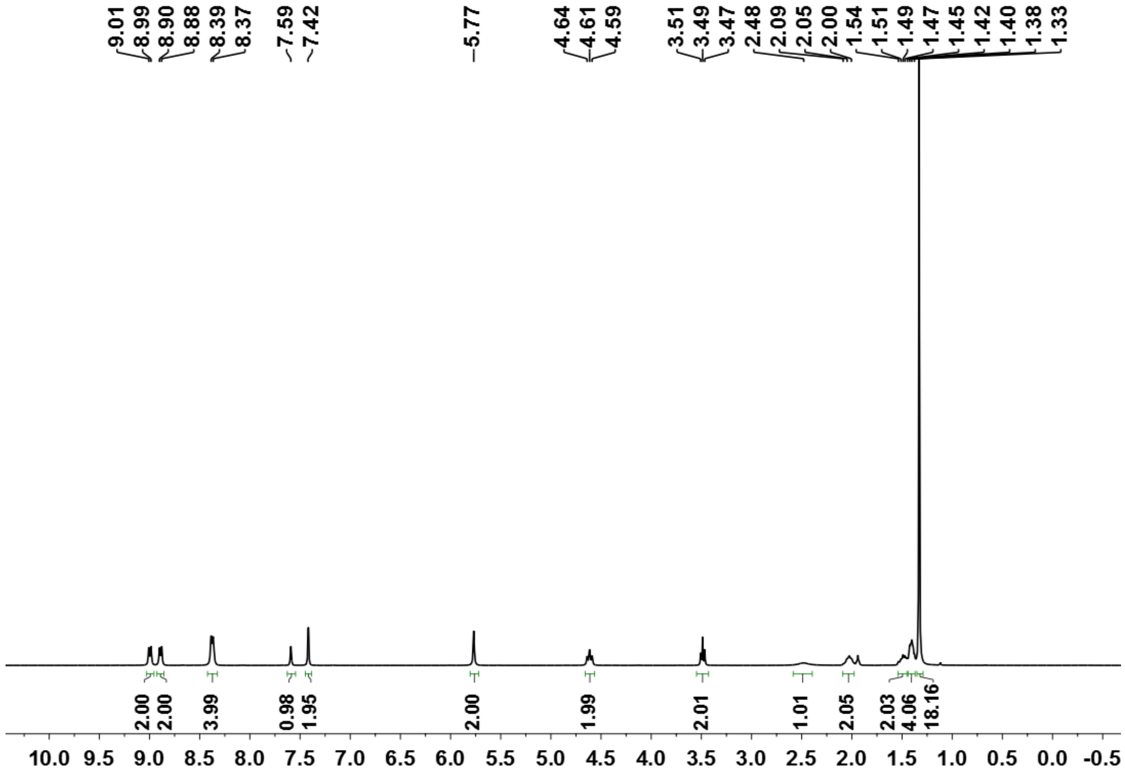

Fig. S7 ^{1}H NMR spectrum (300 MHz, CD_3CN) of **G2**.

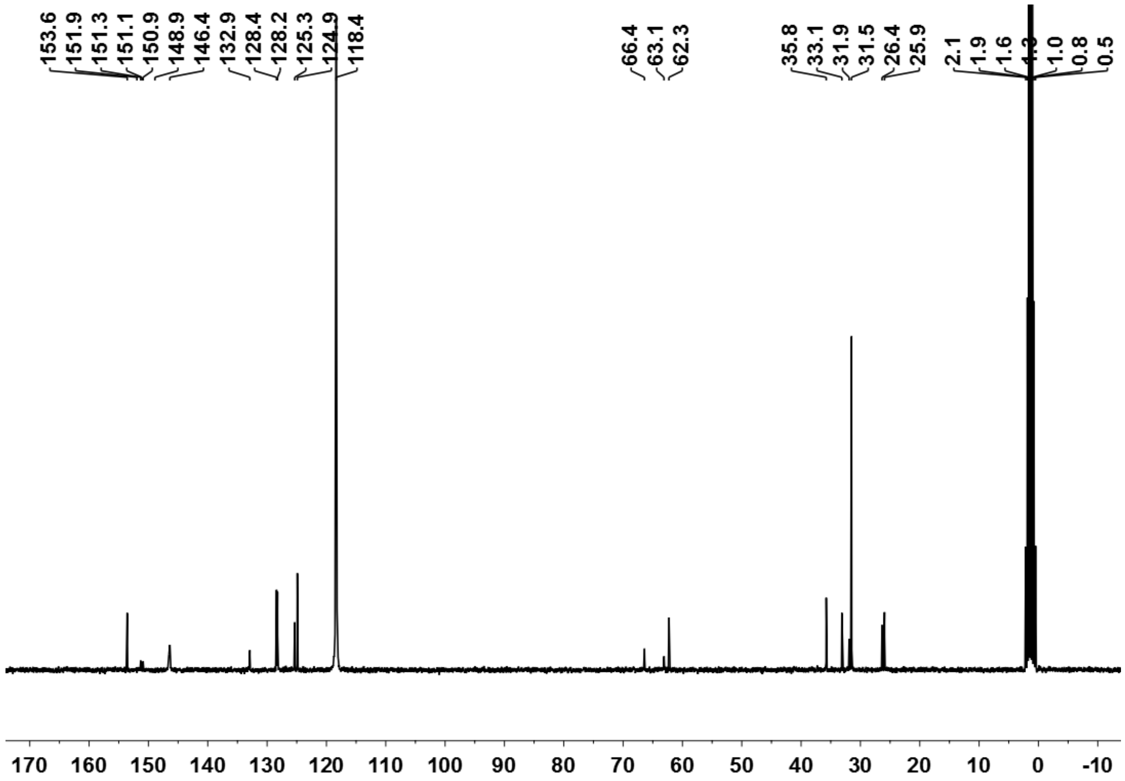

Fig. S8 ^{13}C NMR spectrum (75 MHz, CD_3CN) of **G2**.

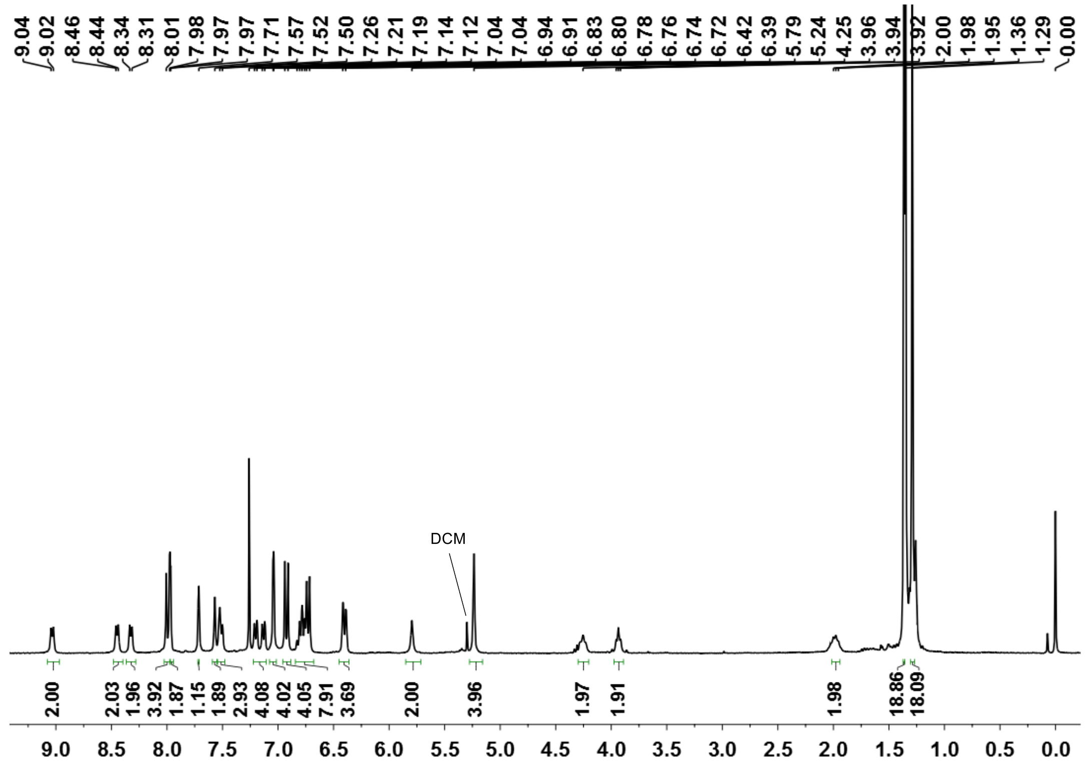

Fig. S9 ^{1}H NMR spectrum (300 MHz, $CDCl_3$) of **R1a**.

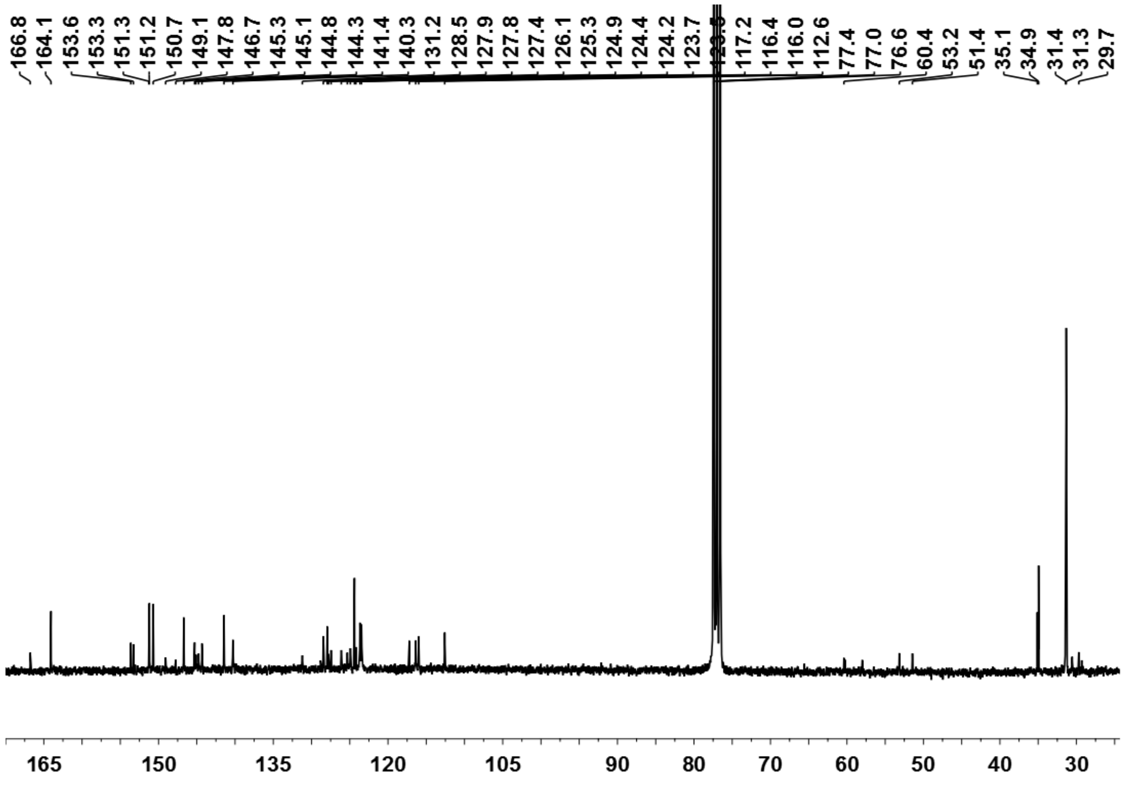

Fig. S10 ^{13}C NMR spectrum (75 MHz, $CDCl_3$) of **R1a**.

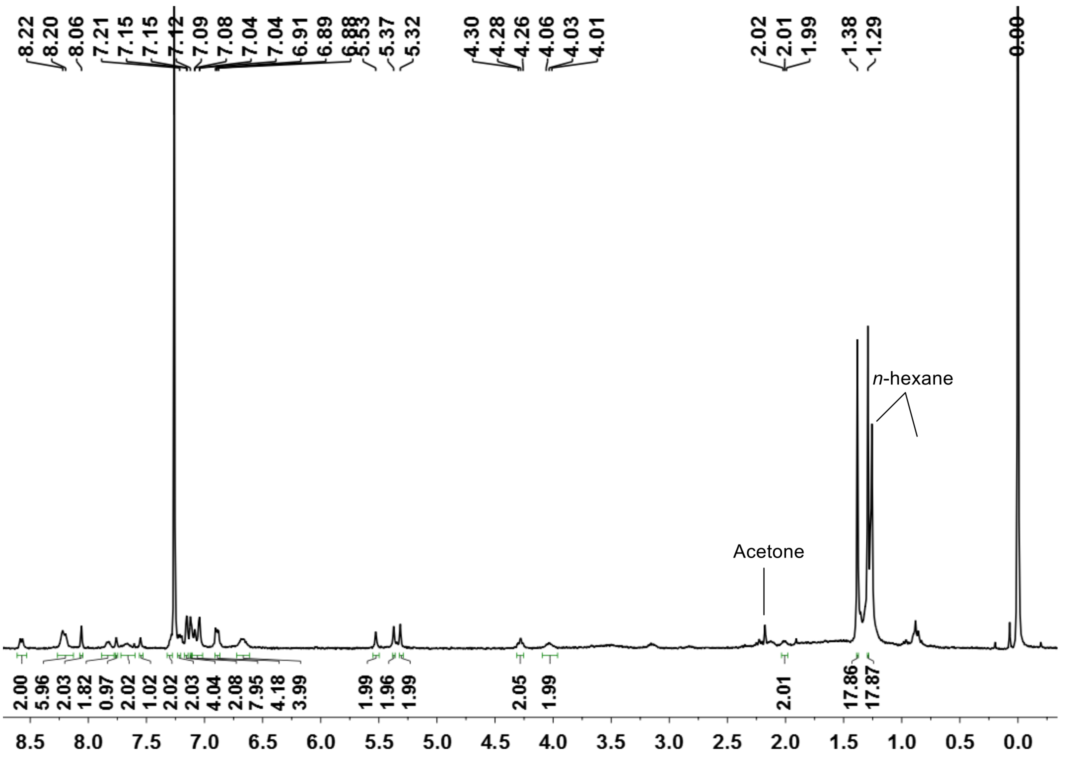

Fig. S11 ^{1}H NMR spectrum (300 MHz, $CDCl_3$) of **R1b**.

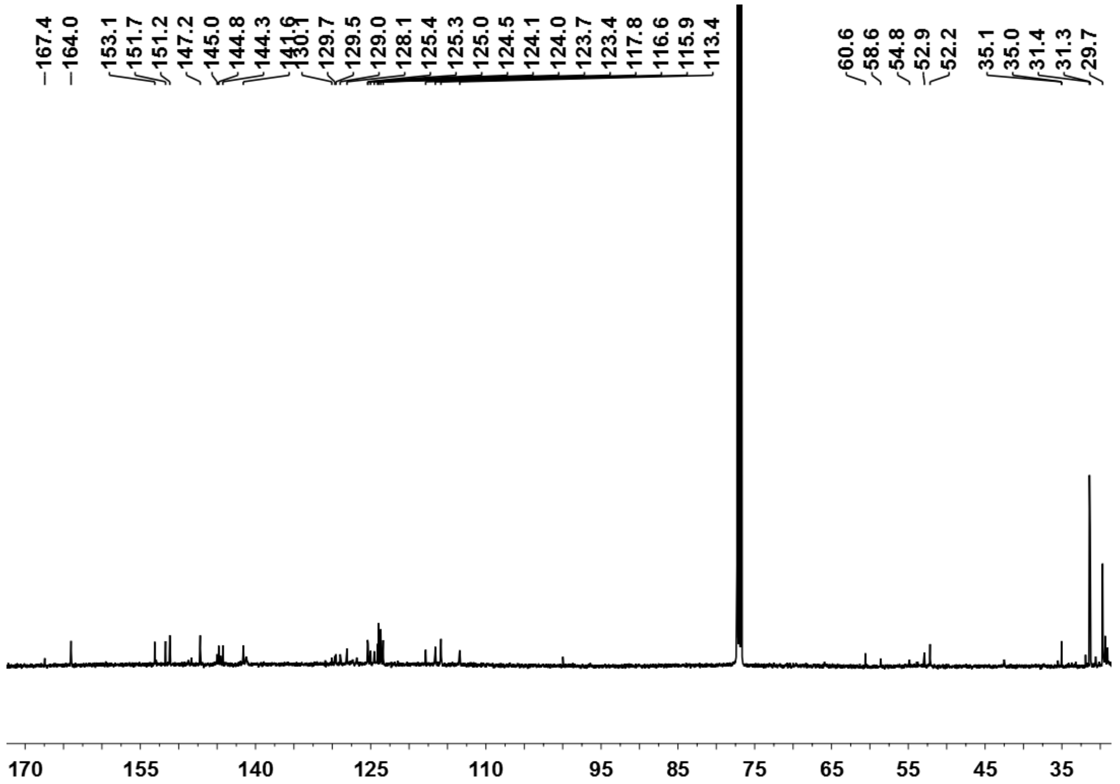

Fig. S12 ^{13}C NMR spectrum (125 MHz, $CDCl_3$) of **R1b**.

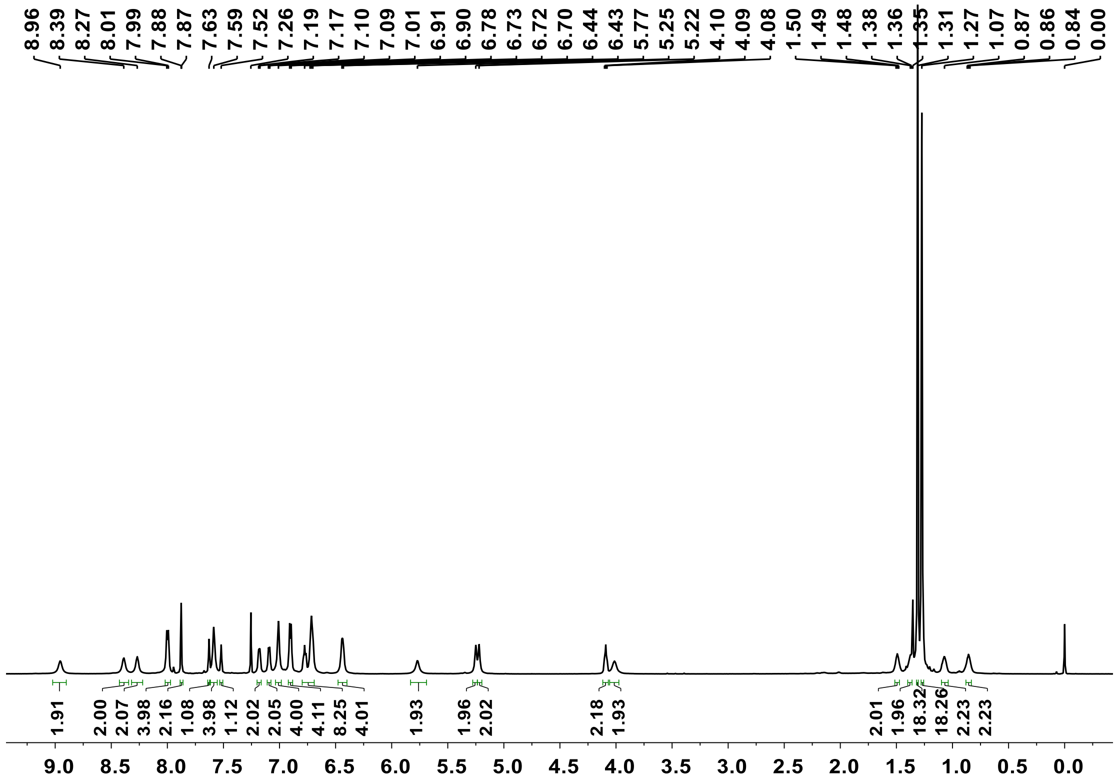

Fig. S13 ^{1}H NMR spectrum (600 MHz, $CDCl_3$) of **R2a**.

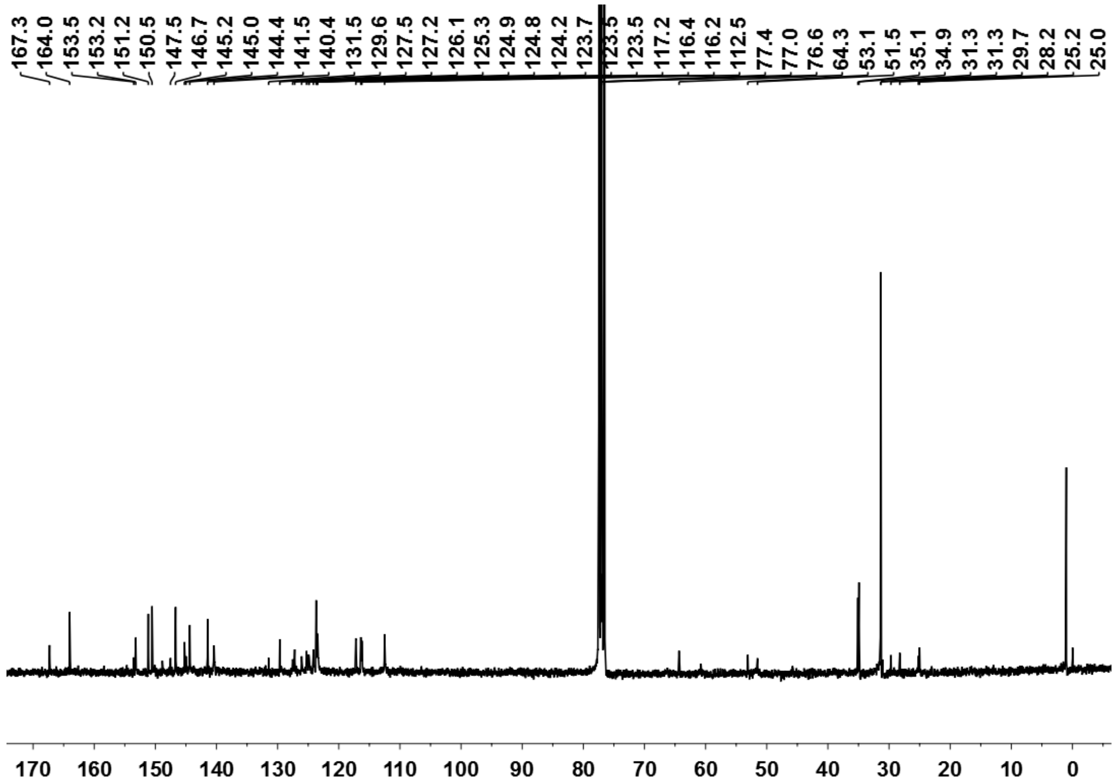

Fig. S14 ^{13}C NMR spectrum (75 MHz, $CDCl_3$) of **R2a.**

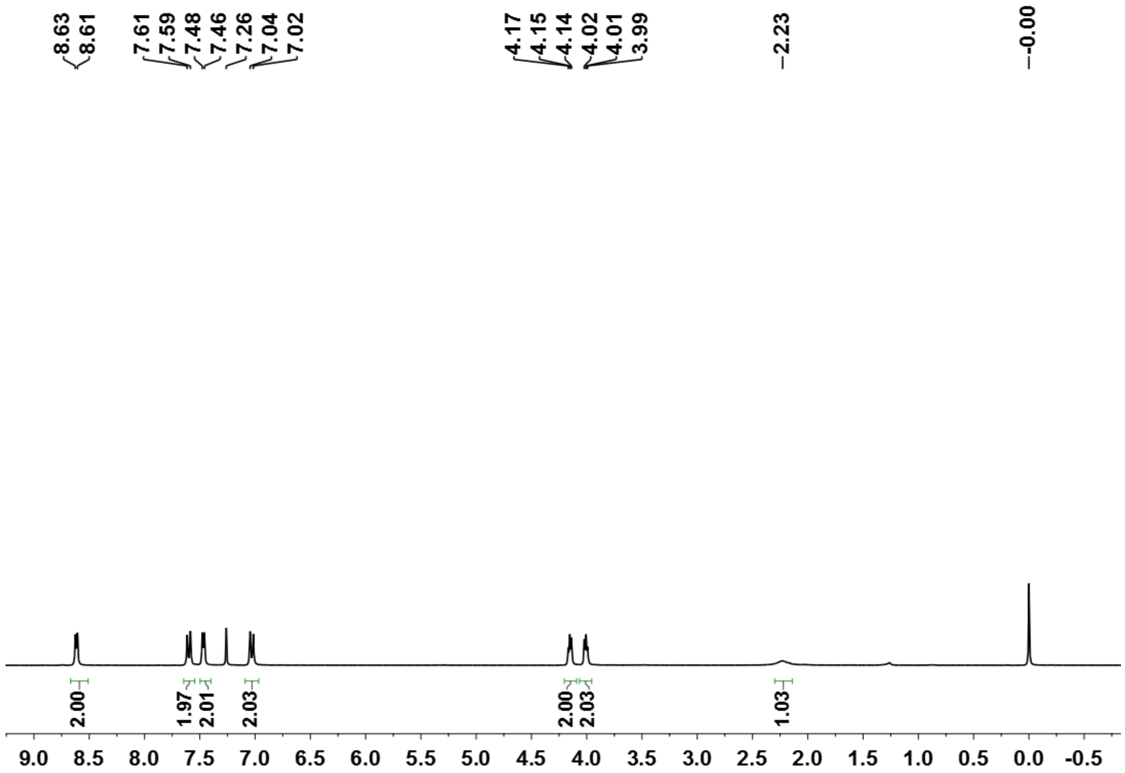

Fig. S15 ^{1}H NMR spectrum (300 MHz, $CDCl_3$) of **3**.

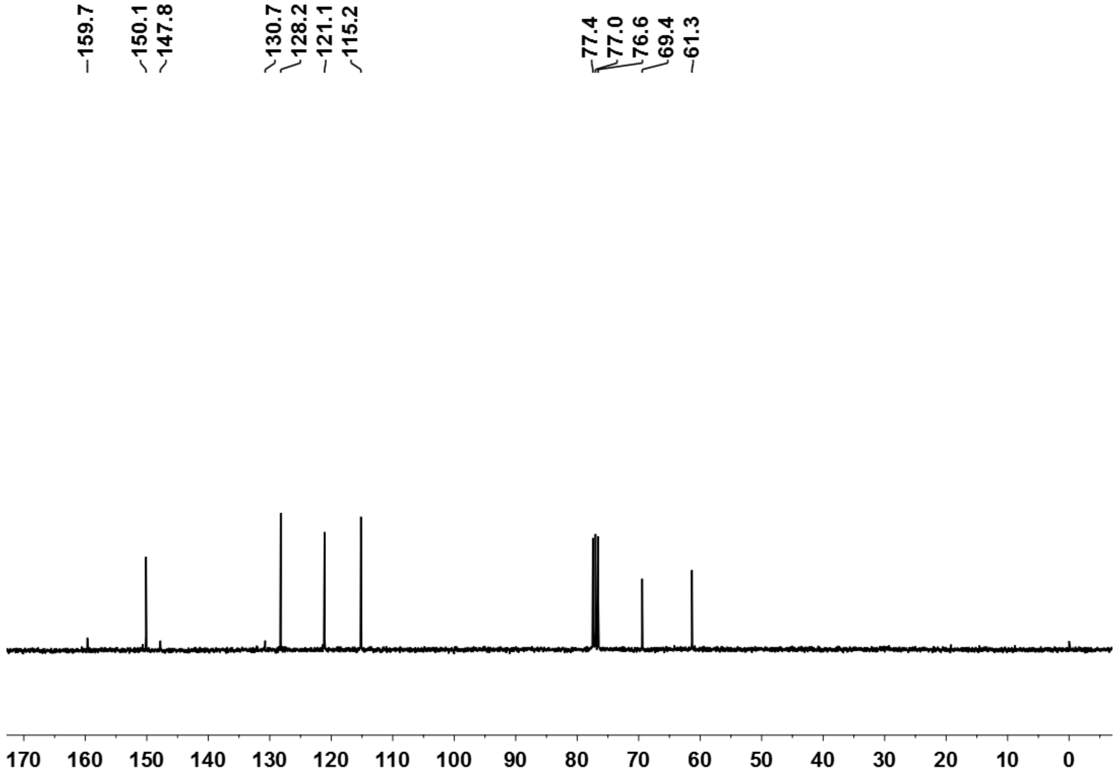

Fig. S16 ^{13}C NMR spectrum (75 MHz, $CDCl_3$) of **3**.

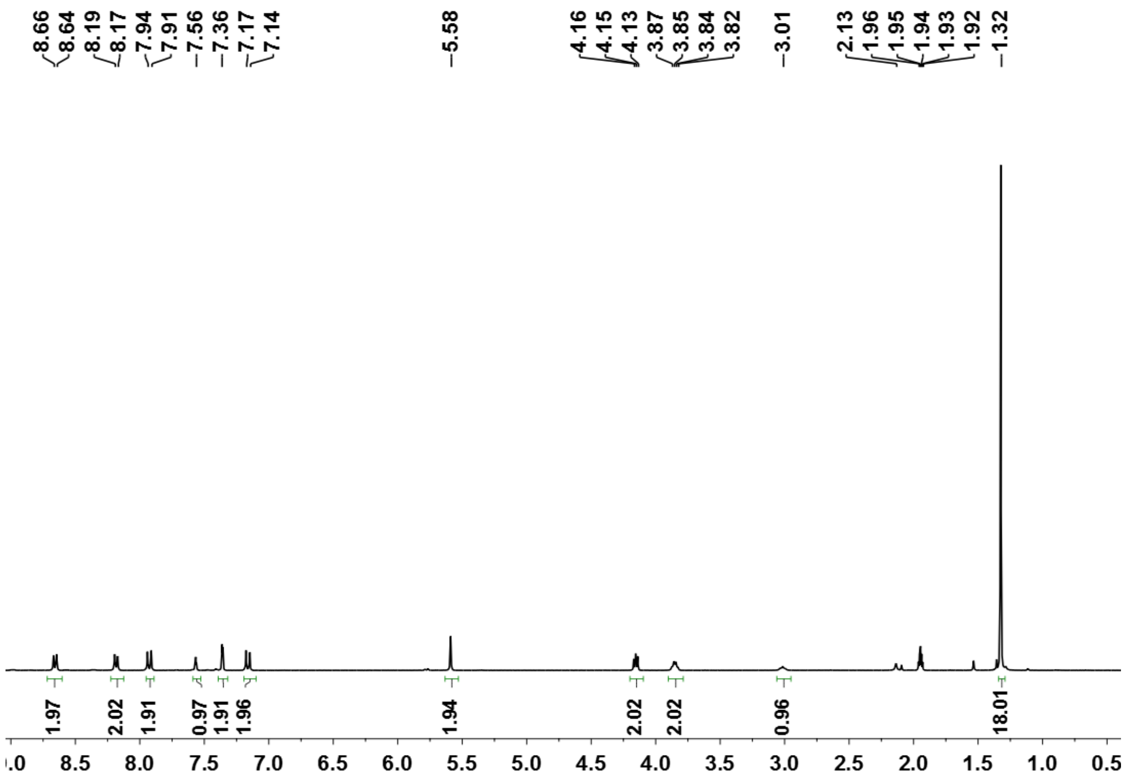

Fig. S17 ^{1}H NMR spectrum (300 MHz, CD_3CN) of **G3**.

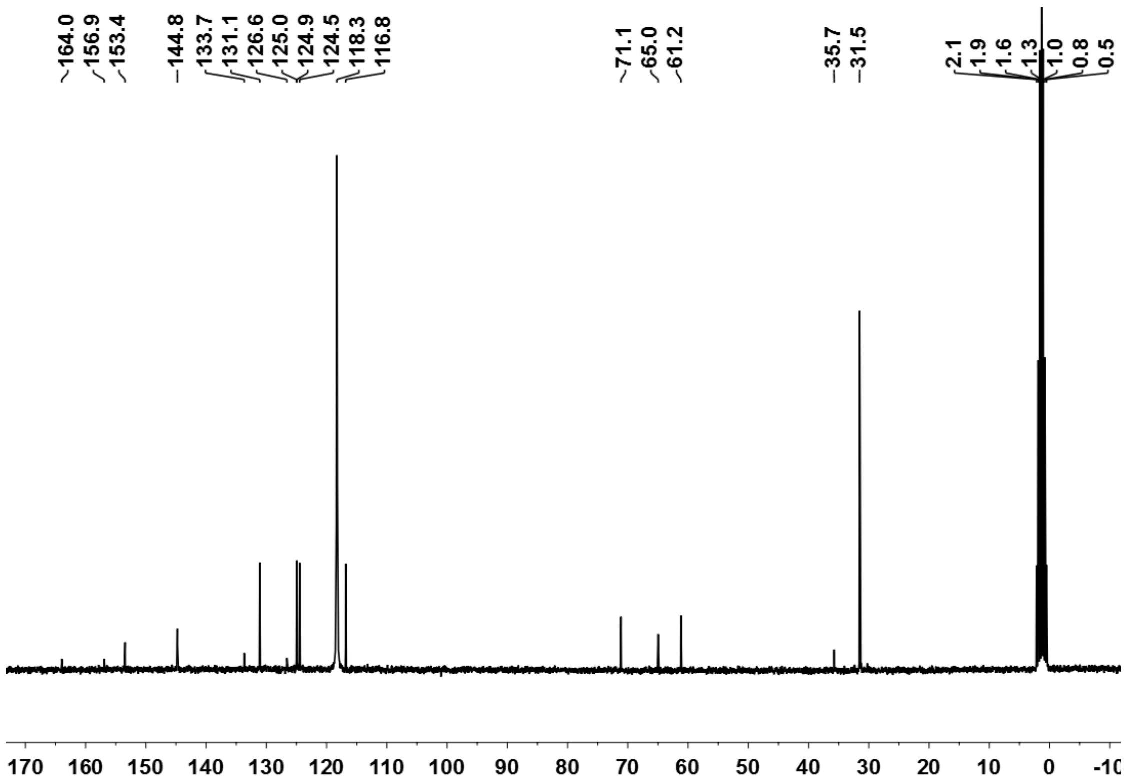

Fig. S18 ^{13}C NMR spectrum (75 MHz, CD_3CN) of **G3**.

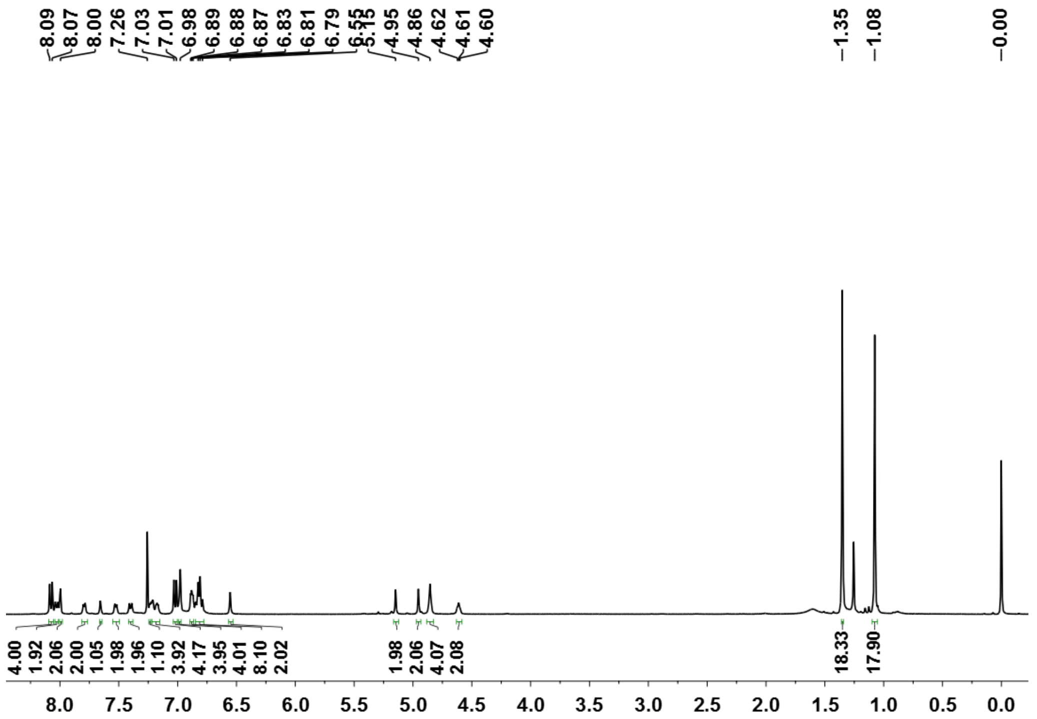

Fig. S19 ^{1}H NMR spectrum (400 MHz, $CDCl_3$) of **R3b**.

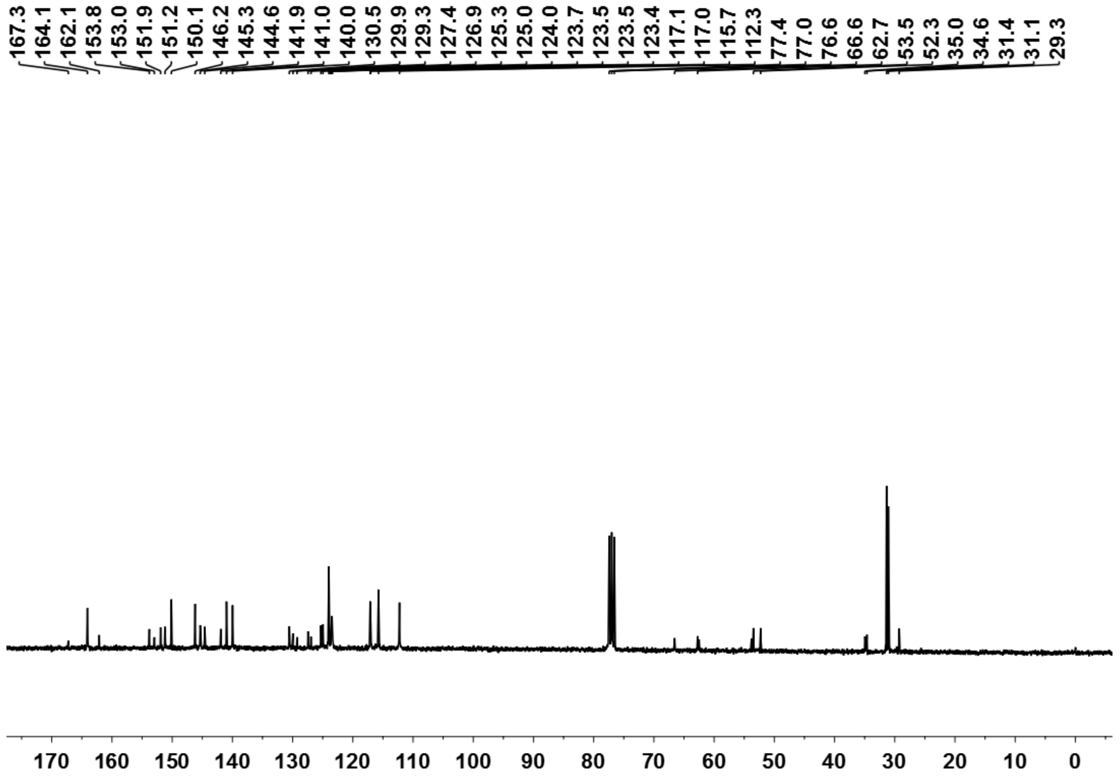

Fig. S20 ^{13}C NMR spectrum (75 MHz, $CDCl_3$) of **R3b**.

3. Copies of 2D NMR spectra of pseudorotaxanes and rotaxanes

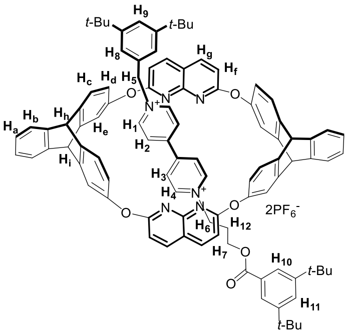

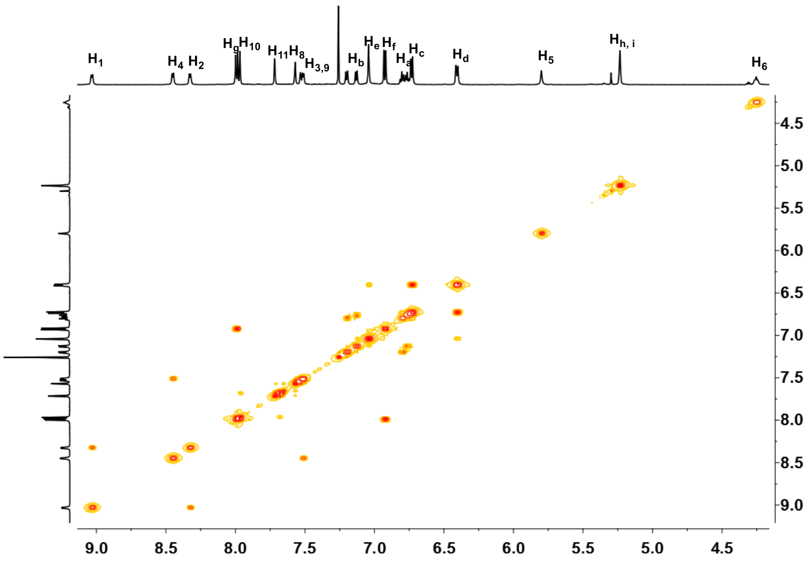

Fig. S21 Partial COSY spectrum (600 MHz, $CDCl_3$) of [2]rotaxane **R1a**.

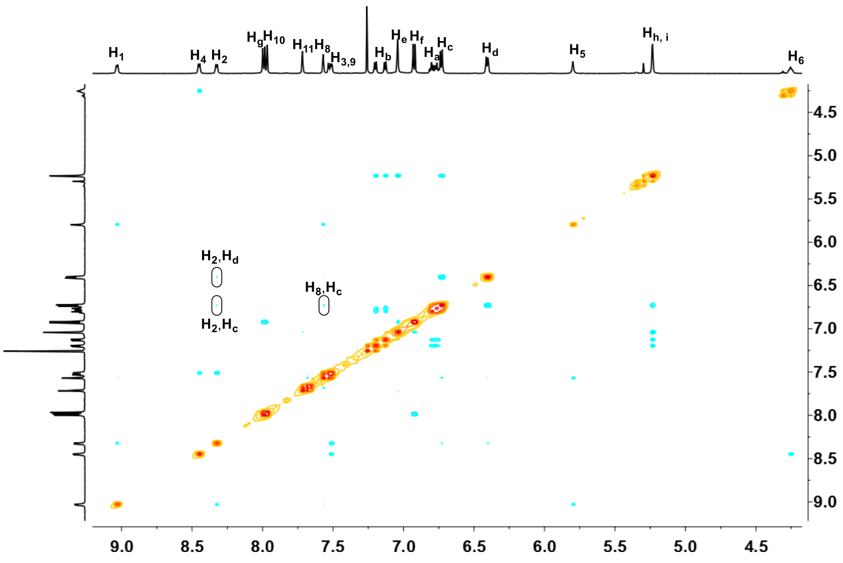

Fig. S22 Partial ROESY spectrum (600 MHz, $CDCl_3$) of [2]rotaxane **R1a**. (correlations denoting the orientation of the host and the guest were marked).

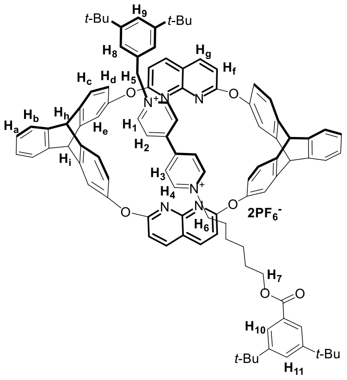

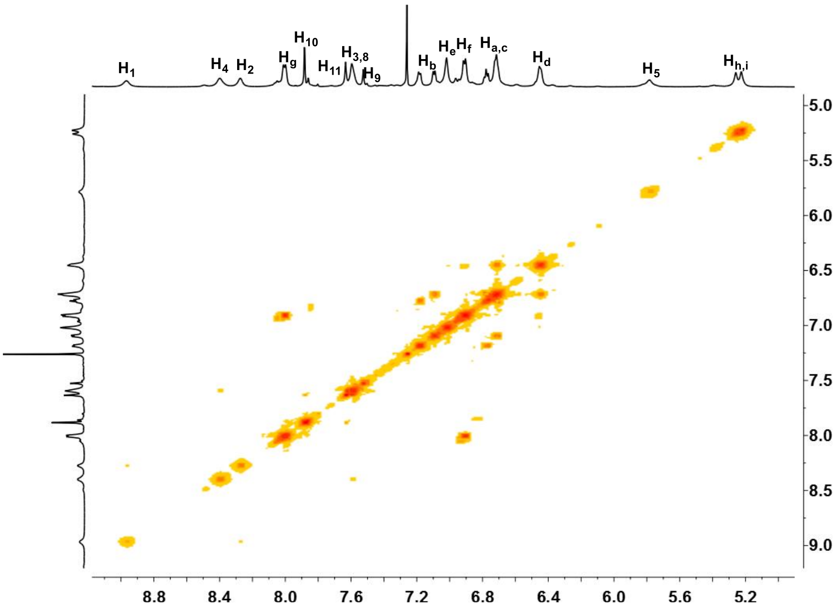

Fig. S23 Partial COSY spectrum (600 MHz, $CDCl_3$) of [2]rotaxane **R2a**.

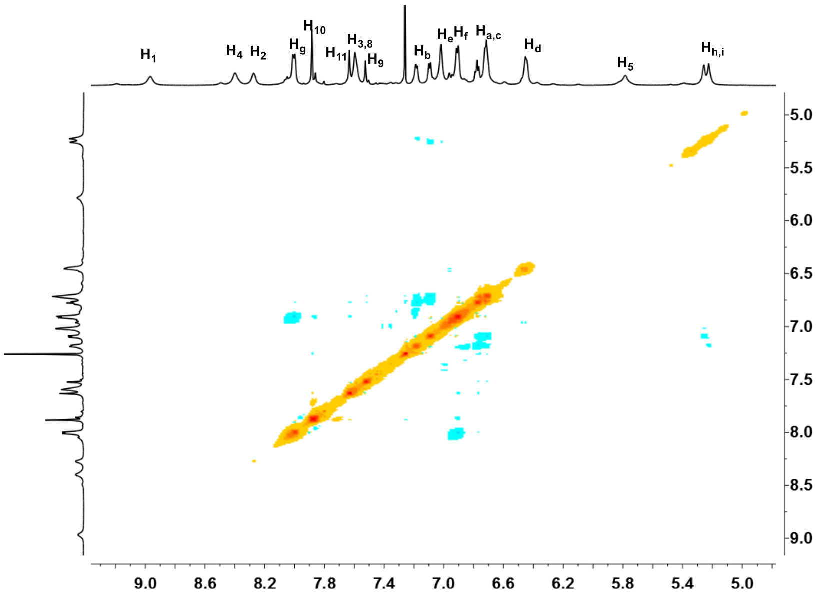

Fig. S24 Partial ROESY spectrum (600 MHz, $CDCl_3$) of [2]rotaxane **R2a**.

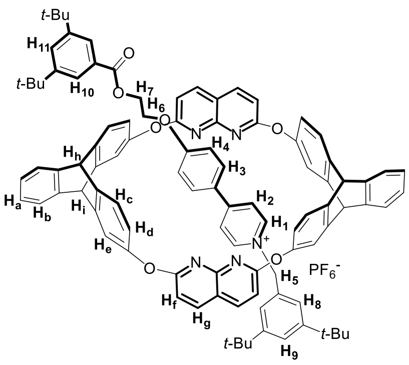

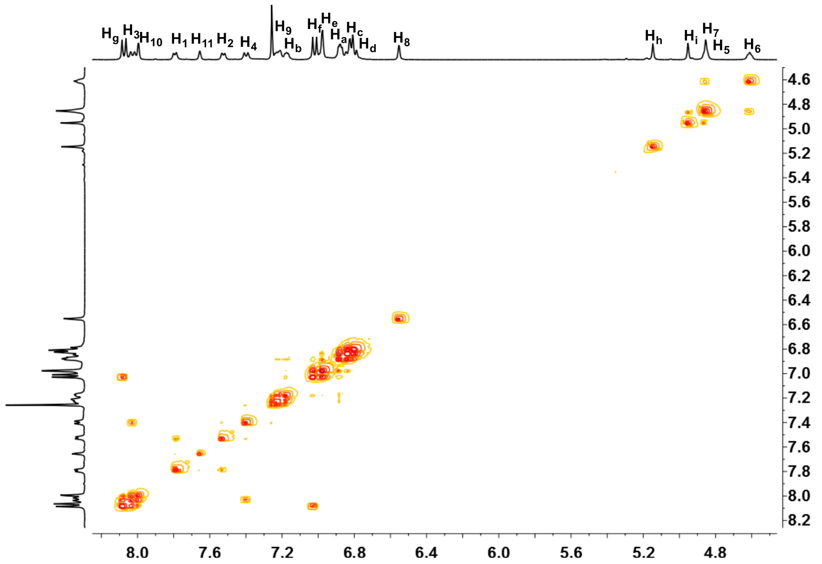

Fig. S25 Partial COSY spectrum (500 MHz, $CDCl_3$) of [2]rotaxane **R3b**.

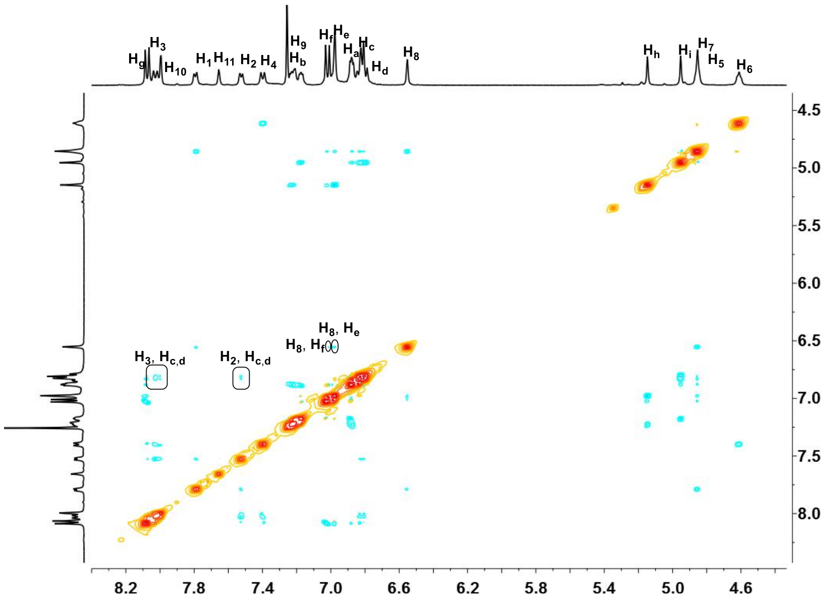

Fig. S26 Partial ROESY spectrum (500 MHz, $CDCl_3$) of [2]rotaxane **R3b** (correlations denoting the orientation of the host and the guest were marked).

4. **Copies of partial ^{1}H NMR spectra showing the ratios of isomers of R1a-b or R2a-b synthesized under different temperatures**

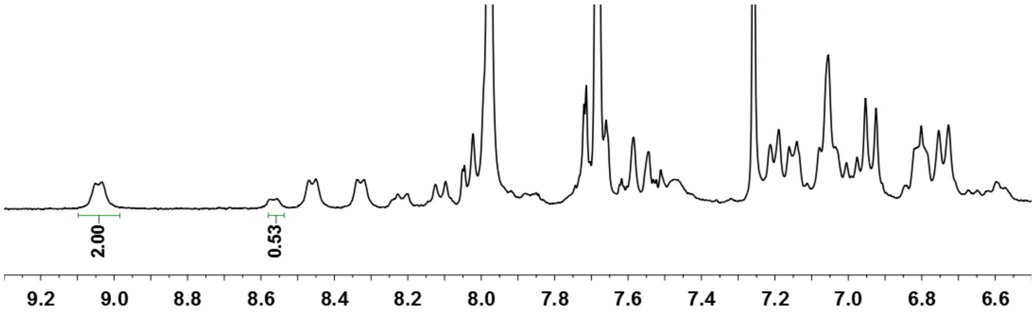

Fig. S27 Partial ^{1}H NMR spectrum (300 MHz, $CDCl_3$) of the crudely isolated[4] mixture of isomers **R1a** and **R1b** synthesized under 298 K.

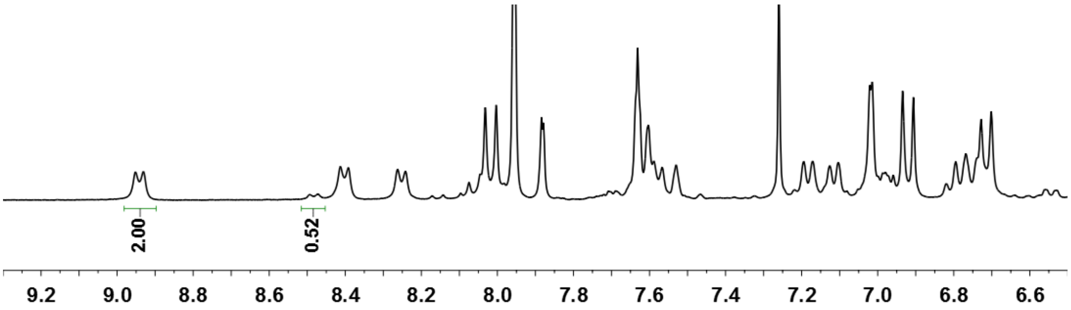

Fig. S28 Partial ^{1}H NMR spectrum (300 MHz, $CDCl_3$) of the crudely isolated mixture of isomers **R2a** and **R2b** synthesized under 298 K.

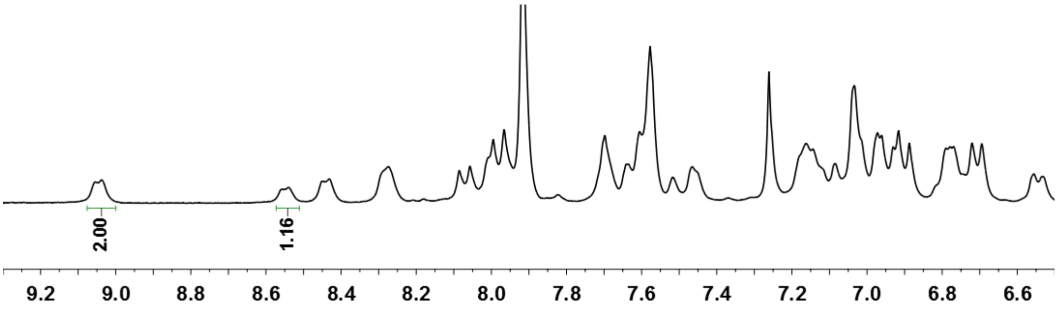

Fig. S29 Partial ^{1}H NMR spectrum (300 MHz, $CDCl_3$) of the crudely isolated mixture of isomers **R1a** and **R1b** synthesized under 313K.

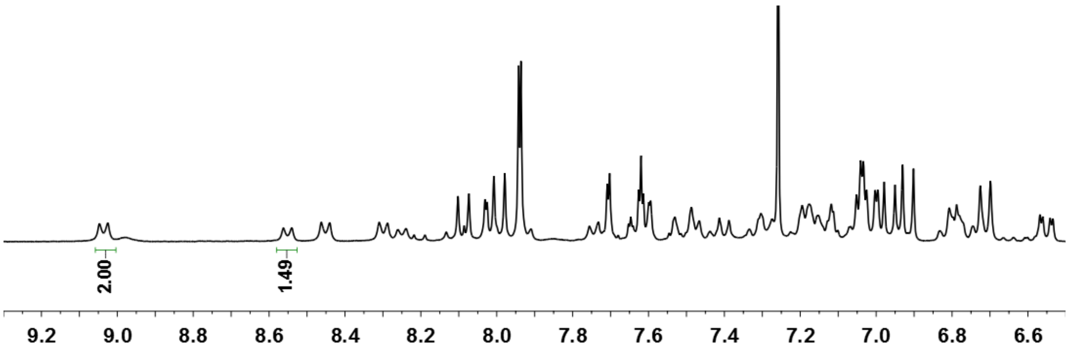

Fig. S30 Partial ^{1}H NMR spectrum (300 MHz, $CDCl_3$) of the crudely isolated mixture of isomers **R1a** and **R1b** synthesized under 333K.

5. ^{1}H NMR spectra of complexation between host H and guests G1-G3

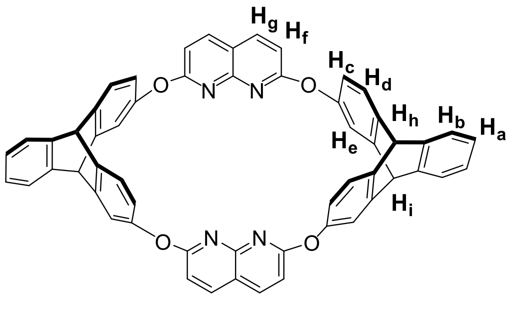

H1

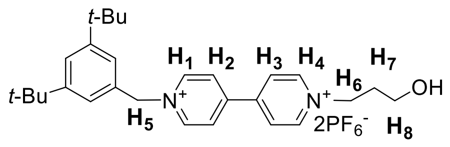

G1

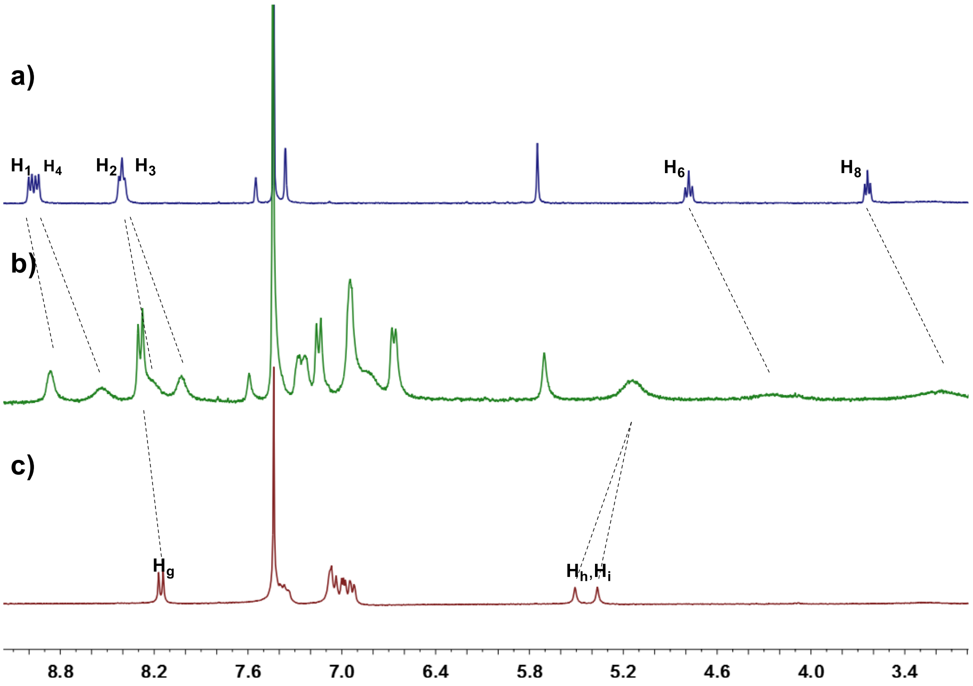

Fig. S31 Partial ^{1}H NMR spectra (300 MHz, $CDCl_3/CD_3CN$, 2:1 *v/v*, 298K) of (a) free host **H**, (b) **H** and 1.0 equiv of **G1**, (c) free guest **G1**. [**H**]$_0$ = 3.0 mM.

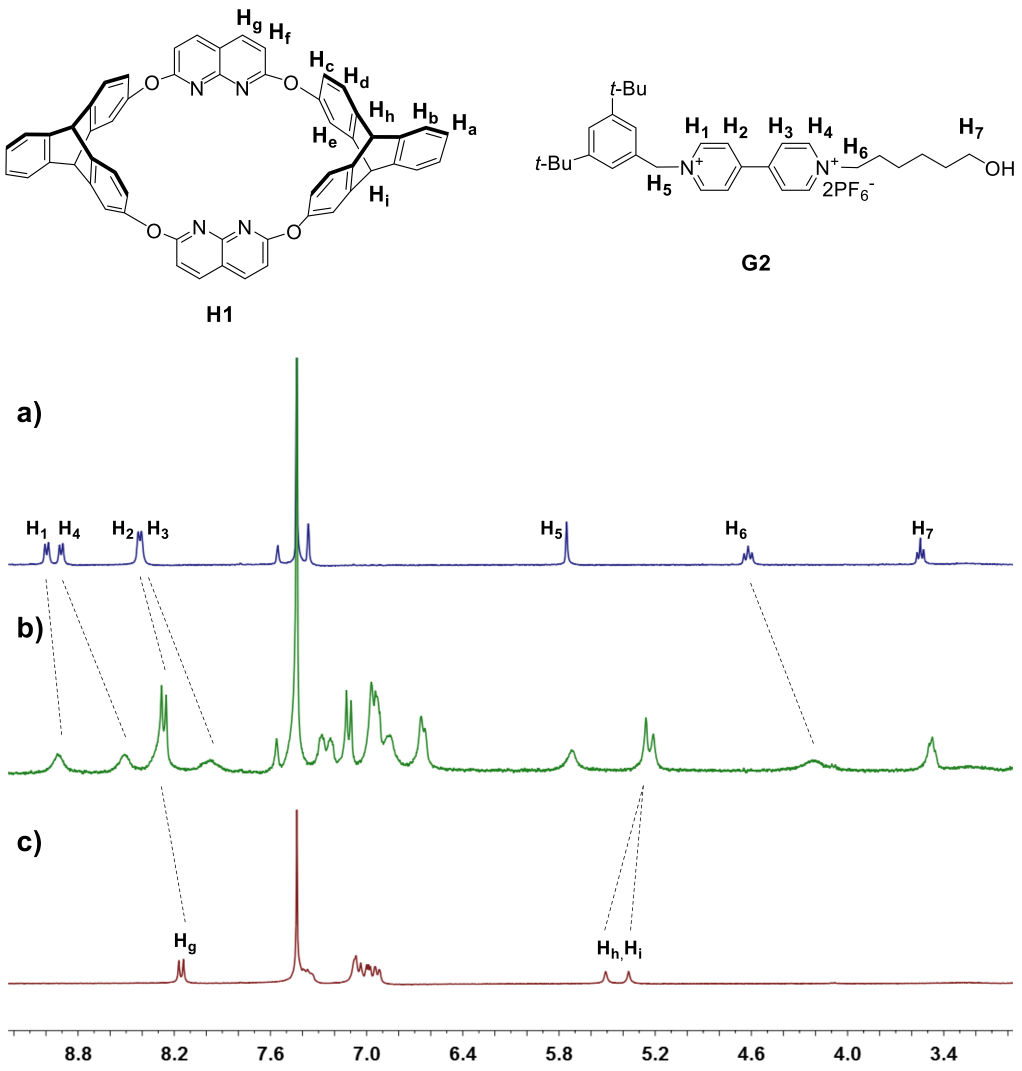

Fig. S32 Partial ^{1}H NMR spectra (300 MHz, $CDCl_3/CD_3CN$, 2:1 *v/v*, 298K) of (a) free host **H**, (b) **H** and 1.0 equiv of **G2**, (c) free guest **G2**. $[\mathbf{H}]_0$ = 3.0 mM.

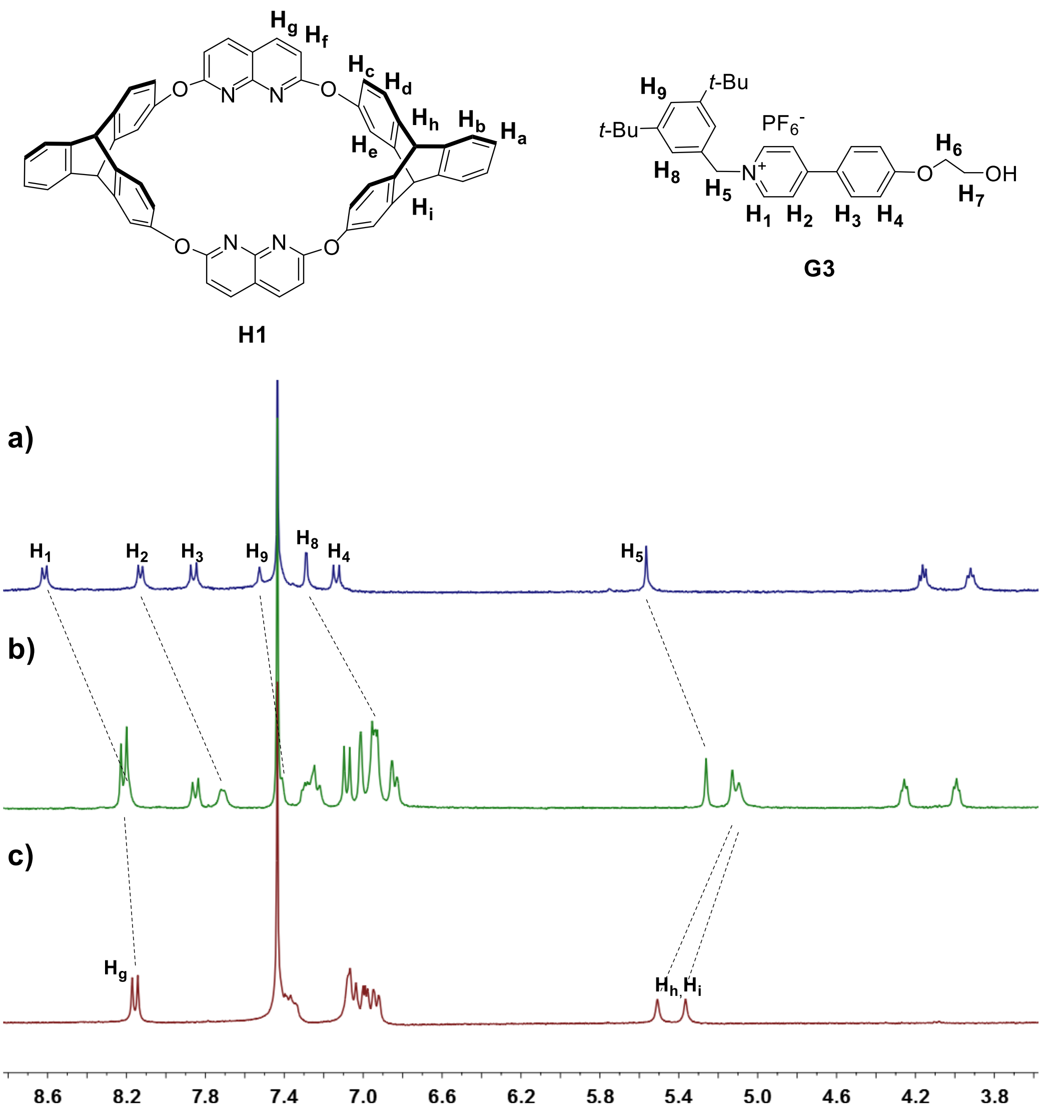

Fig. S33 Partial ^{1}H NMR spectra (300 MHz, $CDCl_3/CD_3CN$, 2:1 *v/v*, 298K) of (a) free host **H**, (b) **H** and 1.0 equiv of **G3**, (c) free guest **G3**. [**H**]$_0$ = 3.0 mM.

6. Determination of the stoichiometries and the association constants

To determine the stoichiometries and association constants of **H@G1**, **H@G2** and **H@G3**, ^{1}H NMR titrations were done. By a nonlinear curve-fitting method, the association constants between the guests and the host were calculated. By a mole ratio plot, the stoichiometry was determined.

The non-linear curve-fitting was based on the equation:

$\Delta\delta=(a/[\mathbf{H}]_0)*(0.5*[\mathbf{G}]_0+0.5*([\mathbf{H}]_0+1/K_a)-(0.5*([\mathbf{G}]_0^2+(2*[\mathbf{G}]_0*(1/K_a-[\mathbf{H}]_0))+(1/K_a+[\mathbf{H}]_0)^2)^{0.5}))$ (Eq. S1)

Where $\Delta\delta$ is the chemical shift change of H_d or H_h on **H** at $[\mathbf{G}]_0$, a is the chemical shift change of H_h when the host iscompletely complexed, $[\mathbf{H}]_0$ is the fixed initial concentration of the host, and $[\mathbf{G}]_0$ is the varying concentration of guests **G1-G3**.

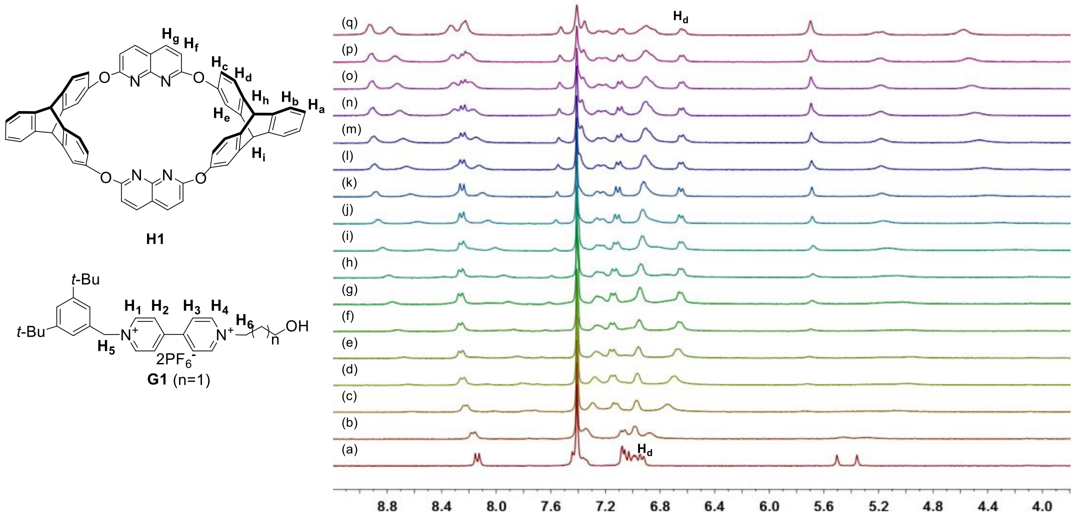

Fig. S34 ^{1}H NMR spectra (300 MHz , $CDCl_3/CD_3CN$, 2:1 *v/v*, 298K) of **H** at a concentration of 3.00 mM with different concentrations of **G1**: (a) 0.00 mM; (b) 1.08 mM; (c) 1.62 mM; (d) 2.16 mM; (e) 2.70 mM; (f) 3.24 mM; (g) 3.78 mM; (h) 4.86 mM; (i) 5.40 mM; (j) 6.48 mM; (k) 7.56 mM; (l) 8.64 mM; (m) 9.72 mM; (n) 10.80 mM; (o) 11.88 mM; (p) 16.20 mM; (q) 21.60 mM.

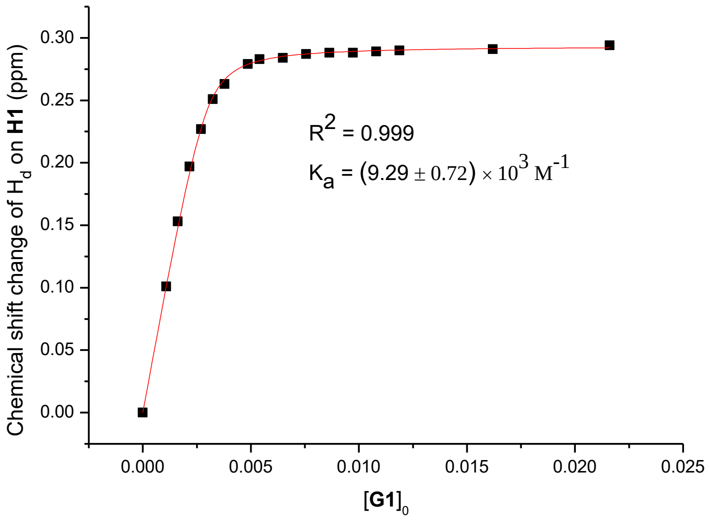

Fig. S35 The chemical shift changes of H_d on **H** upon the addition of **G1**. The red solid line was obtained from the non-linear curve-fitting using Eq. S1.

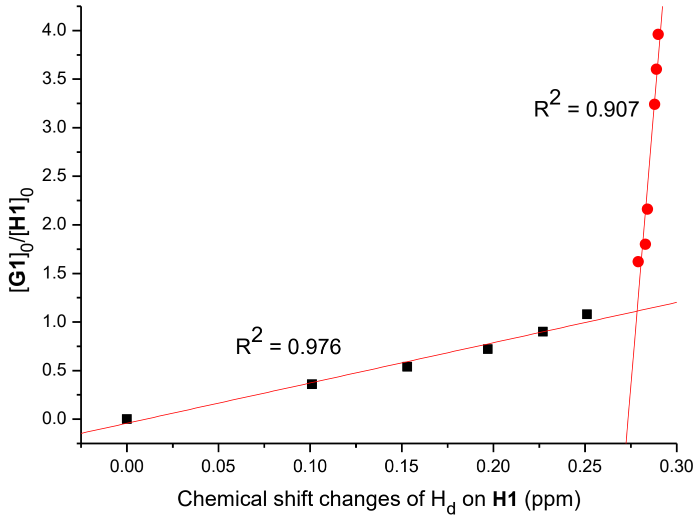

Fig. S36 Mole ratio plot for the complexation between **H** and **G1**, indicating a 1:1 stoichiometry.

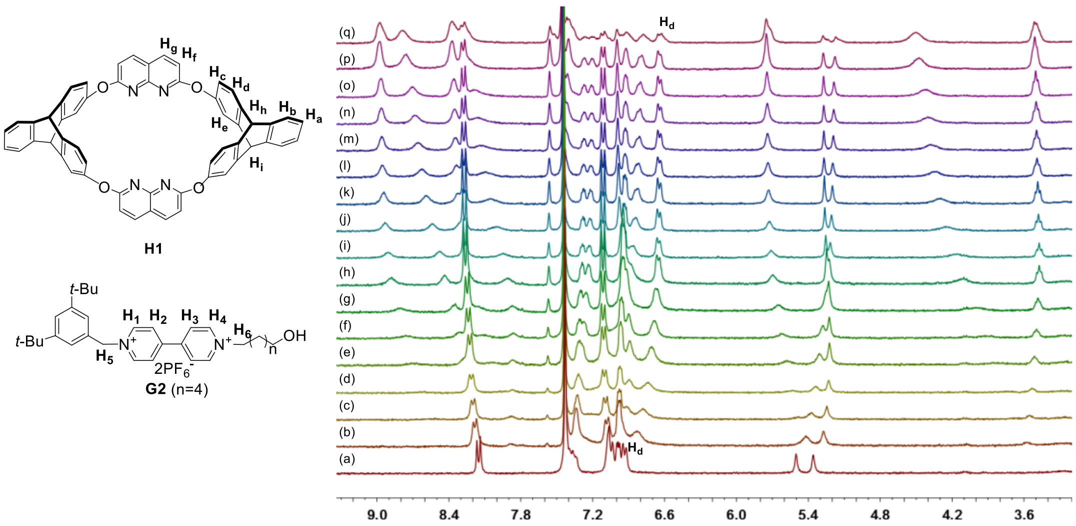

Fig. S37 ^{1}H NMR spectra (300 MHz , $CDCl_3/CD_3CN$, 2:1 *v/v*, 298K) of **H** at a concentration of 2.80 mM with different concentrations of **G2**: (a) 0.00 mM; (b) 0.98 mM; (c) 1.47 mM; (d) 1.96 mM; (e) 2.45 mM; (f) 2.93 mM; (g) 3.42 mM; (h) 4.40 mM; (i) 4.89 mM; (j) 5.87 mM; (k) 6.85 mM; (l) 7.82 mM; (m) 8.80 mM; (n) 9.78 mM; (o) 10.76 mM; (p) 14.67 mM; (q) 19.56 mM.

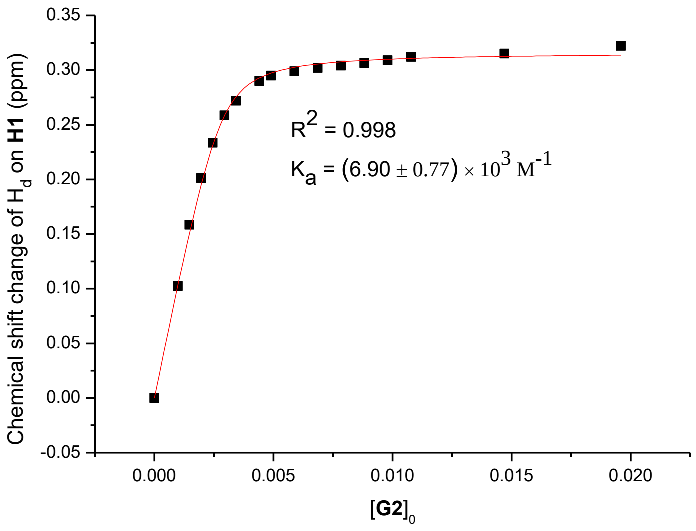

Fig. S38 The chemical shift changes of H_d on **H** upon the addition of **G2**. The red solid line was obtained from the non-linear curve-fitting using Eq. S1.

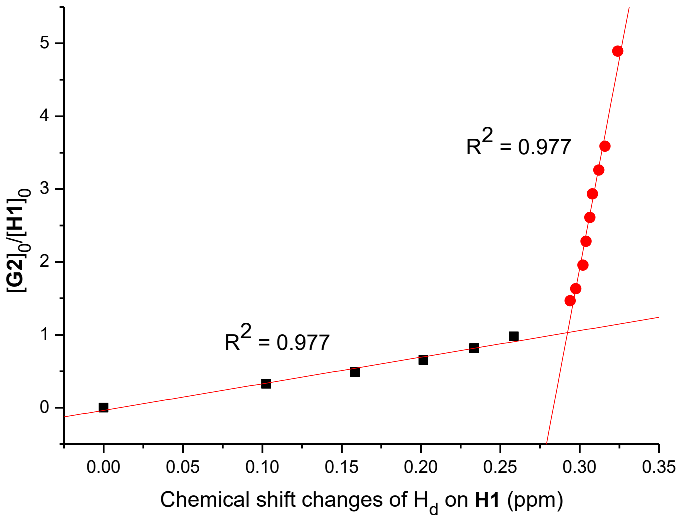

Fig. S39 Mole ratio plot for the complexation between **H** and **G2**, indicating a 1:1 stoichiometry.

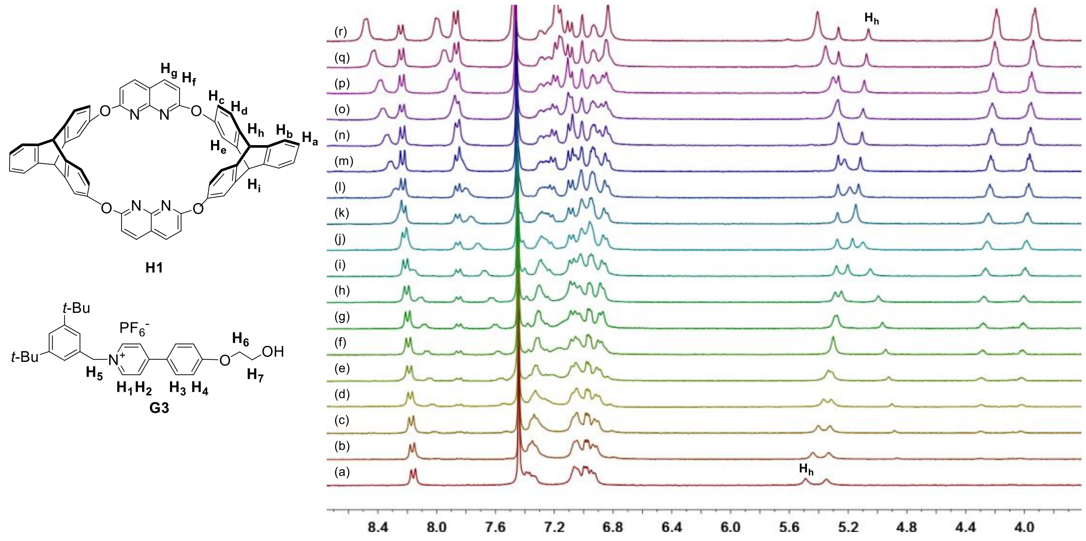

Fig. S40 ^{1}H NMR spectra (300 MHz , $CDCl_3/CD_3CN$, 2:1 *v/v*, 298K) of **H** at a concentration of 3.00 mM with different concentrations of **G3**: (a) 0.00 mM; (b) 0.60 mM; (c) 0.90 mM; (d) 1.20 mM; (e) 1.50 mM; (f) 1.80 mM; (g) 2.10 mM; (h) 2.40 mM; (i) 3.00 mM; (j) 3.60 mM; (k) 4.20 mM; (l) 4.80 mM; (m) 5.40 mM; (n) 6.00 mM; (o) 6.60 mM; (p) 7.20 mM; (q) 9.00 mM.

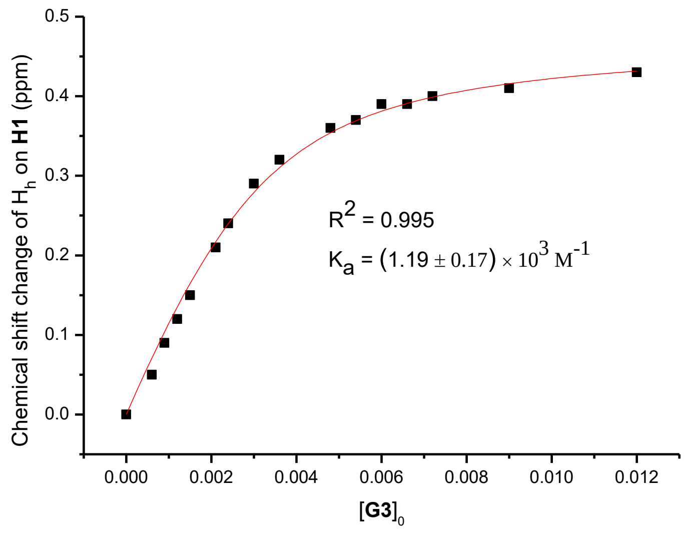

Fig. S41 The chemical shift changes of H$_h$ on **H** upon the addition of **G3**. The red solid line was obtained from the non-linear curve-fitting using Eq. S1.

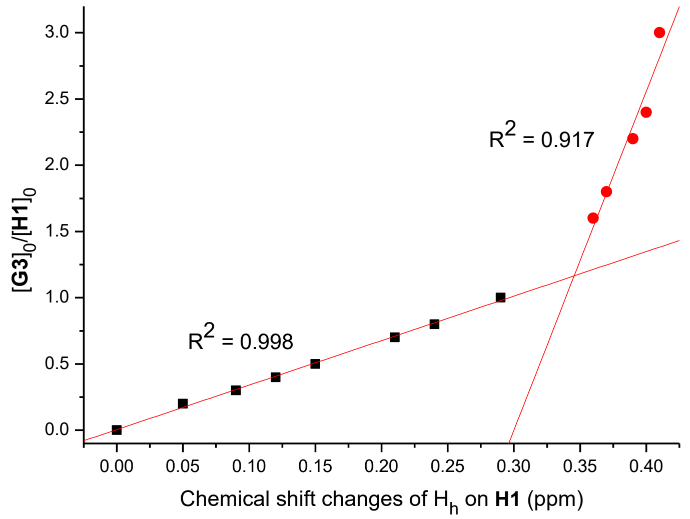

Fig. S42 Mole ratio plot for the complexation between **H** and **G3**, indicating a 1:1 stoichiometry.

7. Variable-temperature ^{1}H NMR spectra of H@G3 and G3.

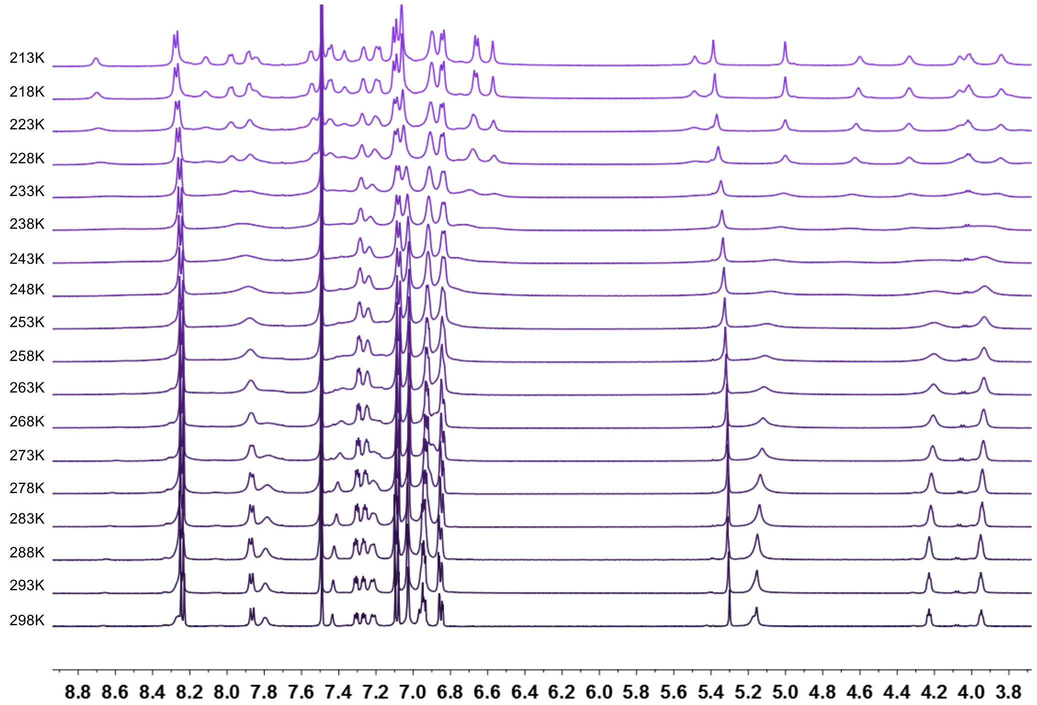

Fig. S43 Variable-temperature ^{1}H NMR spectra of **H@G3** (1:1.5, 500 MHz, $CDCl_3/CD_3CN$, 1:1 *v/v*). [**H**]$_0$= 3.0 mM.

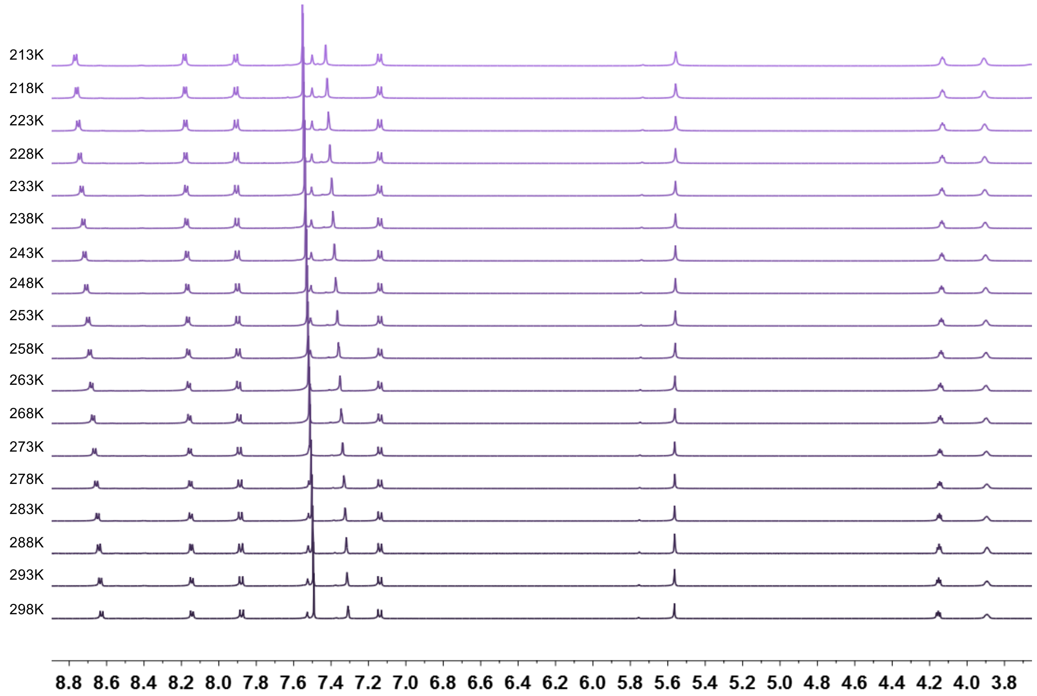

Fig. S44 Variable-temperature ^{1}H NMR spectra of **G3** (500 MHz, $CDCl_3/CD_3CN$, 1:1 *v/v*). [**G3**]$_0$ = 4.5 mM.

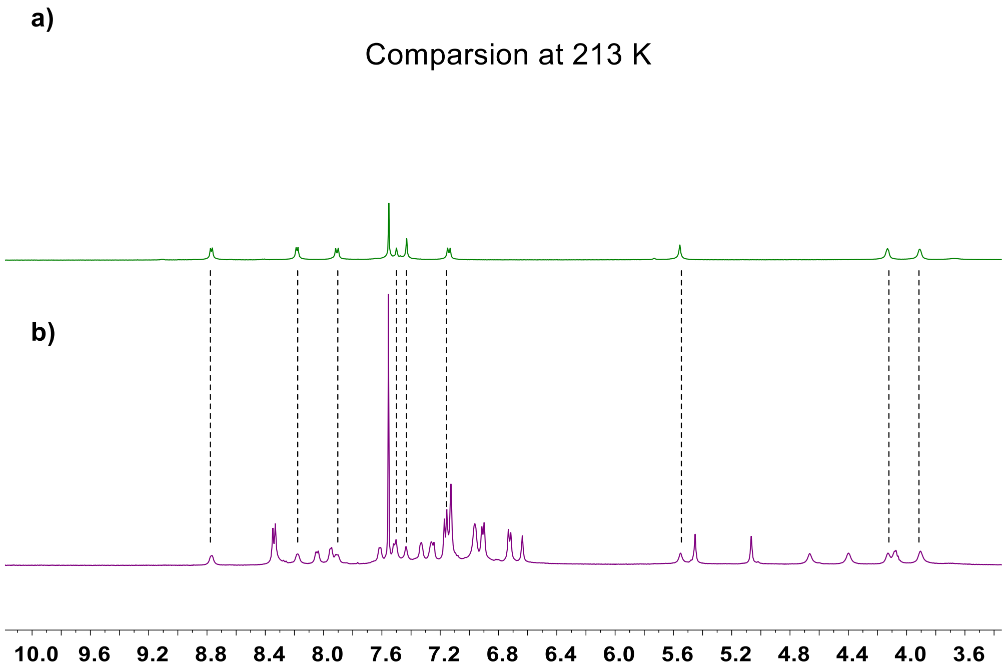

Fig. S45 Comparsion of ^{1}H NMR spectra of a) **G3** (500 MHz, $CDCl_3/CD_3CN$, 1:1 *v/v*), and b) **H@G3** (1:1.5, 500 MHz, $CDCl_3/CD_3CN$, 1:1 *v/v*) at 213K. [**H**]$_0$ = 3.0 mM, [**G3**]$_0$ = 4.5 mM.

7. Crystal data

X-ray structural determination of pseudorotaxane **PR1a**, rotaxanes **R1a** and **R3b**: CCDC 1009829 (**PR1a**), CCDC 1009830 (**R1a**) and CCDC 1419743 (**R3b**) contain the supplementary crystallographic data for this paper. These data can be obtained free of charge via www.ccdc.cam.ac.uk/conts/retrieving.html (or from the Cambridge Crystallographic Data Centre, 12 Union Road, Cambridge CB21EZ, UK; fax: +44 1223 336 033; or deposit@ccdc.cam.ac.uk).

Crystal data for PR1a: $C_{86}H_{74}Cl_4F_{12}N_6O_5P_2$, M_w = 1703.25, monoclinic, space group *P 21/n*, a =11.1928(19) Å, b = 21.893(4) Å, c = 32.961(6) Å, α = 90°, β = 92.044(3)°, γ = 90°, V = 8072(2) $Å^3$, Z = 4, D = 1.402 Mg m^{-3}, T = 173(2) K, 54637 reflections measured, 18426 unique (R_{int} = 0.0616), final R indices [I>2σ(I)]: R_1 = 0.1285, wR_2 = 0.3020, R indices (all data): R_1 = 0.1526, wR_2 = 0.3181.

Crystal data for R1a: $C_{106}H_{97}Cl_{21}F_{12}N_6O_6P_2$, M_w = 2585.28, triclinic, space group *P-1*, a =11.354(2) Å, b = 17.925(4) Å, c = 30.835(6) Å, α = 103.44(3)°, β = 93.42(3)°, γ = 103.76(3)°, V = 5885(2) $Å^3$, Z = 2, D = 1.459 Mg m^{-3}, T = 173(2) K, 52898 reflections measured, 26219 unique (R_{int} = 0.0690), final R indices [I>2σ(I)]: R_1 = 0.1055, wR_2 = 0.2671, R indices (all data): R_1 = 0.1317, wR_2 = 0.2908.

Crystal data for R3b: $C_{99}H_{88}F_6N_5O_7P$, *Mw* = 1604.71, tetragonal, space group *I* 41/a, a = 33.379(5) Å, b = 33.379(5) Å, c = 40.257(8) Å, α = 90°, β = 90°, γ = 90°, V = 44854(16) $Å^3$, Z = 16, D = 0.951 Mg m^{-3}, T = 173(2) K, 136082 reflections measured, 20189 unique (R_{int} = 0.0715), final R indices [I>2σ(I)]: R_1 = 0.1781,wR_2 = 0.4830, R indices (all data): R_1 =0.1898, wR_2 = 0.4905. [5]

9. Computational methods

All DFT calculations were carried out with Gaussian 09 program[6] using B3LYP hybrid functional[7]. Geometries were fully optimized in gas phase consistently employing polarized double-ζ def2-SVP basis set[8] on all atoms, with Grimme's DFT-D3[9] empirical dispersion correction (with zero short range damping). Energies in chloroform solution were calculated as single-point energies from optimized structures using larger polarized triple-ζ def2-TZVP basis set[7] and SMD continuum solvation field.[10] Reported energies (in kcal/mol) are Gibbs free energies, including thermal free energy correction, solvent effect correction, and DFT-D3 empirical dispersion correction.

10. Cartesian Coordiates of Computed Structures

H@G1 (major isomer, PR1a)

O	11.88461900	6.35427600	17.83524000
H	11.69097500	5.59557700	17.26757500
N	9.98842300	7.53043700	19.78186000
N	4.38801200	3.29180100	19.71961400
C	11.33272500	7.52300400	17.25461500
H	11.79154500	7.75839100	16.27523900
H	10.24966500	7.39956800	17.06974500
C	11.54934800	8.69489900	18.20470200
H	10.93686600	9.54418900	17.86673800
H	12.60110400	9.02187500	18.18808300
C	11.22241900	8.35068000	19.66297900
H	12.02933300	7.75766400	20.10631200
H	11.06040100	9.25222200	20.26703100
C	10.08981700	6.24349600	20.18434400
H	11.08768000	5.89077900	20.43750000
C	8.98277300	5.41883900	20.23108200
H	9.10376000	4.38680700	20.56734000
C	7.73531600	5.92673500	19.82670300
C	7.65871300	7.26961100	19.42703600
H	6.73113900	7.71582000	19.06955600
C	8.80001100	8.05935800	19.41500300
H	8.79694200	9.11075900	19.10121000
C	6.55913000	5.03124600	19.79316500
C	6.73542300	3.66073900	19.53659100
H	7.72667400	3.23604400	19.37073900
C	5.63788600	2.82400000	19.50094300
H	5.73408700	1.75286400	19.32776200
C	4.18901400	4.60288200	19.98020200
H	3.16086700	4.91379100	20.16889100
C	5.25129700	5.49340200	20.01533500
H	5.05196300	6.53794300	20.25688400
C	3.24597100	2.30818000	19.78356800
H	2.32239700	2.90059300	19.78131500
H	3.28366700	1.72415400	18.85371400
C	3.36992200	1.43769900	21.00824100
C	4.06396800	0.22418000	20.93513500
H	4.41352600	-0.13579700	19.96409300
C	4.31100600	-0.53590100	22.08856600
C	3.81468100	-0.04401300	23.30720700
H	3.99731300	-0.61925000	24.20949500
C	3.08929600	1.15536200	23.41343600
C	2.87829500	1.89034700	22.23716000
H	2.32323800	2.83120400	22.27724400
C	5.12277100	-1.84001600	21.98256600
H	5.79053900	-1.93055300	24.07866700
C	2.53076500	1.67361300	24.75180400
C	0.99063400	1.76096400	24.65020900
H	0.66650300	2.45606400	23.85990600
H	0.56614500	2.11917900	25.60120700
H	0.55245200	0.77461800	24.43248400
C	3.11294300	3.07486800	25.04627800

H	4.20947100	3.04446900	25.13084400
H	2.71432100	3.45936300	25.99769000
H	2.85231900	3.80640700	24.26411300
C	2.89135500	0.75160500	25.92970400
H	2.47486700	-0.25949100	25.80382900
H	2.47525800	1.16257100	26.86162600
H	3.98086700	0.66612600	26.06572900
O	8.92045800	11.84082000	16.69348000
O	9.57829100	10.04281400	23.32876600
O	8.72661100	1.08021500	22.76679200
O	10.47527300	3.30228700	16.41998400
N	9.17125900	11.27745500	18.91156800
N	9.38517300	10.68399700	21.12555100
N	9.31777100	1.81227200	20.65572100
N	9.88494600	2.54326400	18.53744900
C	9.25326600	12.17543800	17.95843700
C	9.69782300	13.51511700	18.14810800
H	9.73505200	14.18994700	17.29266800
C	10.05901400	13.89493100	19.41753000
H	10.40818100	14.91121200	19.61705200
C	9.97029300	12.95867000	20.48488300
C	10.31204000	13.23584400	21.83551000
H	10.67366600	14.23047800	22.10855700
C	10.17357900	12.25386800	22.78902600
H	10.40816600	12.41205200	23.84174400
C	9.68747100	10.98693600	22.36491200
C	9.50557600	11.64304700	20.17541300
C	8.65814700	9.01371600	23.29060900
C	9.04147900	7.84262700	23.96094000
H	10.04662400	7.77792100	24.38242300
C	8.10582100	6.83167100	24.13974300
C	6.79479600	6.96726100	23.64197600
C	6.44115400	8.11481400	22.93811400
H	5.41932400	8.24304800	22.56852700
C	7.37254400	9.14837900	22.75538500
H	7.08529700	10.06250800	22.23767700
C	7.26467600	5.61940800	26.07062900
C	5.95037100	5.76932200	25.59010300
C	4.88058600	5.85041200	26.47504700
H	3.86041400	5.96843600	26.10134300
C	5.12816100	5.77927500	27.85497200
H	4.29536100	5.84407600	28.55908000
C	6.43246800	5.62893100	28.33192000
H	6.61612200	5.57531600	29.40743000
C	7.51160300	5.54877900	27.43755900
H	8.53208900	5.43359600	27.81179000
C	8.30918000	5.55460900	24.94862900
H	9.33323800	5.44413900	25.32616900
C	7.87351300	4.39710000	24.05580300
C	6.54733500	4.52810700	23.59464100
C	5.88002500	5.81727100	24.05977800
H	4.85318600	5.92568900	23.68653300
C	5.95320500	3.49655700	22.87894100
H	4.90760300	3.56502700	22.58049300
C	6.68386800	2.33731800	22.58341500
H	6.22542700	1.50283300	22.05561200
C	8.00716200	2.24500500	23.00284700
C	8.61607800	3.26275200	23.74856600
H	9.64143900	3.13234400	24.10027500
C	9.41956200	0.92472400	21.61764000
C	10.22663600	-0.24811600	21.55761900
H	10.23656100	-0.93052300	22.40764200
C	10.97408200	-0.45144000	20.42575800
H	11.62183300	-1.32677400	20.33247100
C	10.90741100	0.49063300	19.36213300
C	10.03899100	1.61572300	19.51902900
C	11.64252100	0.38243700	18.15285400
H	12.32058100	-0.46184200	18.00471400
C	11.48607500	1.33720900	17.17696400
H	12.01699500	1.30561000	16.22544400
C	10.57027000	2.39519800	17.42769700
C	9.39391300	4.12594100	16.18371800
C	9.68595600	5.27925100	15.43876900
H	10.70881100	5.44838300	15.09481900
C	8.66244400	6.16357700	15.12868200
C	7.34356900	5.91284700	15.55817600
C	7.05741500	4.74199900	16.24951700
H	6.02899800	4.51742800	16.54570900
C	8.08097700	3.83258700	16.55753000
H	7.85805300	2.90155900	17.07110600
C	7.78895500	7.34714800	13.17967200
C	8.06812400	7.46625100	11.82215400
H	9.08914400	7.65284800	11.47954200
C	7.01976000	7.34580400	10.89692700
H	7.22756600	7.43894300	9.82856000
C	5.71407100	7.10976100	11.33429900
H	4.90445100	7.01958300	10.60643400
C	5.43454500	6.98967900	12.70432700

H	4.41214600	6.80733100	13.04569800
C	6.47440300	7.10920300	13.62042700
C	6.36658900	7.00959000	15.14490600
H	5.34071100	6.82157600	15.48758100
C	8.79448800	7.45639300	14.32977300
H	9.81915700	7.64449100	13.98591800
C	8.26109800	8.55633400	15.24417800
C	6.94825800	8.31271800	15.69265000
C	6.30983300	9.23915200	16.51435400
H	5.28062700	9.06954600	16.84306500
C	6.97422400	10.41683900	16.88588600
H	6.48552100	11.16590600	17.51051700
C	8.27217600	10.64462300	16.42637500
C	8.93049100	9.71977400	15.60826500
H	9.93779200	9.94215500	15.25077000
C	6.54343300	-1.50388300	21.46883400
H	6.52278200	-1.01323200	20.48192300
H	7.13712900	-2.42549400	21.36193000
H	7.07309200	-0.83998400	22.16940400
C	5.26143200	-2.55198400	23.33944900
H	5.84179900	-3.47829500	23.21407700
H	4.28245200	-2.83216300	23.75755900
C	4.42426500	-2.80123500	20.99478100
H	3.40467300	-3.04174700	21.33345200
H	4.98837100	-3.74389300	20.91823000
H	4.35256700	-2.37839900	19.98072000

H@G1 (minor isomer, PR1b)

O	24.78671600	17.27544600	28.44559400
N	21.19461500	19.17223700	22.46467900
C	22.92168200	22.74589600	20.32654000
C	22.07258700	22.13257000	21.25239700
H	22.07974300	22.43601000	22.29839300
C	21.21787900	21.08978200	20.86255500
C	21.19220000	20.66125500	19.53466700
H	20.52773200	19.84115400	19.25896900
C	22.02386900	21.25499600	18.56843400
C	22.87327400	22.28526500	18.99468400
H	23.53261400	22.75585000	18.26760900
C	23.91291300	23.85416400	20.72271100
C	23.73527000	24.29136300	22.18825700
H	24.45426900	25.09016700	22.42654400
H	23.91275300	23.46448900	22.89288600
H	22.72659400	24.69364000	22.37319500
C	25.35055300	23.31175800	20.54688900
H	25.54855200	23.00633700	19.50814900
H	25.52380200	22.43894700	21.19771000
H	26.08780100	24.08614800	20.81347400
C	23.70851300	25.08950000	19.81808000
H	24.40810600	25.89045000	20.10430500
H	22.68398500	25.48202200	19.91220300
H	23.88700700	24.86209200	18.75670200
C	22.00311900	20.73202100	17.12128600
C	22.84390700	21.60531600	16.17411200
H	22.77683100	21.21325000	15.14816700
H	23.90965700	21.60899200	16.45069600
H	22.48729800	22.64691800	16.15610100
C	20.54985100	20.70504300	16.59572000
H	20.53160000	20.35174000	15.55287800
H	20.09978900	21.70967800	16.62195600
H	19.90771000	20.02905800	17.18135500
C	22.57742700	19.29535900	17.11307300
H	21.98893500	18.61454800	17.74593600
H	23.61782700	19.28867100	17.47793100
H	22.57650300	18.88920300	16.08876100
C	20.43540000	20.34775200	21.90200000
H	20.20609100	20.97880400	22.77310700
H	19.49556200	19.93908200	21.51781000
C	22.49287000	18.96605000	22.16384600
H	22.93562100	19.63098400	21.42525100
C	23.22375300	17.98441800	22.80503100
H	24.27581100	17.86270800	22.55209200
C	22.61211200	17.20702400	23.79906600
C	21.23511900	17.37513900	24.01134700
H	20.68740300	16.79905900	24.75652500
C	20.54408700	18.35779300	23.32367800
H	19.47196900	18.52447400	23.44290700
C	23.42946700	16.35866800	24.68921100
C	24.73542800	16.75967900	24.98910800
H	25.21502300	17.61776300	24.51723000
C	25.45155500	16.09379300	25.96621900
H	26.45003400	16.41681600	26.25177300
C	23.68517700	14.58912600	26.30658000
H	23.33005600	13.71733400	26.85794600
C	22.92109100	15.21895400	25.34475000
H	21.93352100	14.82094300	25.11078100
C	25.15469700	15.00360100	29.12936300

H	24.06687600	14.84352400	29.21841600	C	19.37839100	20.55955000	29.75085300
H	25.62865700	14.39413400	29.91589500	H	19.54079300	19.66895400	30.36372900
C	25.47184500	16.46960600	29.39723600	C	19.57077700	20.51354600	28.37396000
H	26.56743500	16.63101600	29.34069500	C	18.70504600	20.24388800	25.70959700
H	25.15996100	16.71283200	30.42902500	C	17.72838600	20.24539400	24.71994200
O	28.82275700	16.45626100	23.51929000	H	17.52839300	21.12687700	24.10727400
O	24.32126200	21.58497400	24.63970700	C	16.98089700	19.07828500	24.51594200
O	15.99686800	19.07619700	23.53367700	C	17.18691400	17.93479300	25.28328500
O	20.90784800	16.88678200	19.21336100	H	16.57671000	17.05000400	25.09518700
N	27.37989200	18.20089600	23.90666200	C	18.16929300	17.94633500	26.28580700
N	25.86121700	19.89332500	24.26302800	H	18.32131200	17.06117200	26.90931200
N	17.58157500	18.32097400	22.05593300	C	18.92766500	19.09446300	26.49606600
N	19.21687400	17.56782000	20.62233900	C	16.34654200	18.69434400	22.28637700
C	28.59699000	17.78903300	23.64186300	C	15.32019200	18.73622200	21.29752900
C	29.71634400	18.65022900	23.47450900	H	14.31599700	19.05194200	21.58172900
C	29.49178500	20.00594700	23.54511700	C	15.65836300	18.38484800	20.01396800
H	30.31304500	20.71307800	23.40282800	H	14.91339000	18.41365800	19.21473400
C	28.17865100	20.49435900	23.79075400	C	16.99294400	17.98266200	19.71810900
C	27.81991100	21.86807700	23.86104100	C	17.92693800	17.94615400	20.79988200
H	28.58165100	22.63607800	23.70455900	C	17.46692000	17.62917300	18.42826000
C	26.51935500	22.21759200	24.13724300	H	16.78421900	17.65162200	17.57499600
H	26.19064700	23.25390100	24.21257600	C	18.78309100	17.25499900	18.26441900
C	25.57895800	21.16932000	24.35508300	H	19.20019600	16.96682800	17.29954200
C	27.14116700	19.53130600	23.98590500	C	19.60894600	17.23024600	19.41947600
C	23.35742800	20.87965300	25.33406400	C	21.66060700	16.11279000	20.07225500
C	22.07293800	21.44975200	25.26159700	C	23.04303300	16.14308300	19.82342300
H	21.92945600	22.35631700	24.67042500	H	23.42956000	16.81984600	19.05889200
C	21.03778100	20.88997400	25.99779500	C	23.86776200	15.24745600	20.49028500
C	21.26033900	19.74792000	26.79516300	C	23.34063900	14.33177300	21.42378500
C	22.53031000	19.18859900	26.85753200	C	21.97807400	14.35765800	21.70708900
H	22.72515100	18.31311200	27.48269400	H	21.54837300	13.64327400	22.41523600
C	23.59242500	19.75439800	26.13110200	C	21.12584700	15.25019600	21.03486300
H	24.58608100	19.32072000	26.19341100	H	20.05559400	15.23835300	21.23266500
C	20.01259300	19.29918000	27.55141800	C	24.37278600	13.30845500	21.90397800
H	20.18352400	18.40673000	28.16709500	H	23.95676000	12.57994500	22.61200000
C	19.60672000	21.41434100	26.09477200	C	25.35098300	15.03046700	20.21851100
H	19.43488200	22.30786300	25.48118300	H	25.76804400	15.75571800	19.50868300
C	19.35479800	21.65884400	27.58592200	C	25.45117600	13.58286000	19.71977500
C	18.94752000	22.85305600	28.17150100	C	25.99232600	13.15992500	18.51063400
H	18.77774300	23.74217000	27.55877800	H	26.40977600	13.88374900	17.80594000
C	18.75368800	22.90062800	29.56071500	C	25.99623900	11.78939800	18.20704400
H	18.43156100	23.83268700	30.03059600	H	26.41995700	11.44589100	17.26059100
C	18.96687400	21.76338900	30.34370200	C	25.46293200	10.86429300	19.10752600
H	18.80969000	21.80930800	31.42380700	H	25.47038200	9.79976000	18.86236700

C	24.91669100	11.29306300	20.32757200
H	24.49960300	10.56796700	21.03135500
C	24.91308300	12.65102500	20.62698700
C	25.55759200	14.08334100	22.47215000
C	26.20904000	13.86312000	23.67968400
H	25.86855900	13.06945200	24.35035900
C	27.31897700	14.65351300	24.03112500
H	27.85973900	14.49741700	24.96647400
C	27.74605200	15.65537200	23.16478500
C	27.13904100	15.84368000	21.91450600
H	27.53367900	16.59706400	21.22986000
C	26.05846000	15.04702400	21.56860900
N	24.92087900	15.04749000	26.62868000
C	25.64241800	14.45493700	27.78378300
H	24.86093300	18.20525000	28.70071900
H	25.49914900	13.36615700	27.73488700
H	26.71295400	14.65523500	27.63582200
H	30.69866500	18.22159900	23.27425900

H@G3 (minor isomer, PR3a)

O	12.33895800	7.41721700	16.85056100
H	12.20875000	6.78653100	17.57339600
N	4.36158800	3.26610400	19.71259300
C	11.42456500	8.47328600	17.04092300
H	11.68184300	9.26917400	16.32274700
H	10.39023200	8.15424600	16.82082200
C	11.47480100	9.05441800	18.44818400
H	10.73944400	9.86375800	18.55895900
H	12.47698900	9.44845100	18.66684700
C	10.13176400	6.13928300	20.17384300
H	11.08958300	5.78478600	20.55839500
C	8.99098300	5.36322700	20.26376700
H	9.04941500	4.37972400	20.73185800
C	7.75742100	5.84281000	19.76399200
C	7.71801400	7.13681400	19.20026200
H	6.78768900	7.53717900	18.79249900
C	8.86430600	7.90677000	19.07113500
H	8.79314800	8.89737600	18.62513700
C	6.58703300	4.98291100	19.76405700
C	6.72775200	3.57597600	19.66892900
H	7.71439300	3.11691900	19.61424300
C	5.62311400	2.76368900	19.63229700
H	5.70918100	1.68053000	19.56179100
C	4.18626100	4.60817600	19.81725600
H	3.15581800	4.95693600	19.89737200
C	5.25925700	5.47483600	19.82895700
H	5.06672900	6.54287000	19.93118900
C	3.20493100	2.32208700	19.76899800
H	2.29350300	2.93244900	19.72932800
H	3.24101300	1.70820300	18.85730700
C	3.25509000	1.46623800	21.01272300
C	3.88088800	0.21526600	20.97681200
H	4.23611300	-0.17464000	20.01949600
C	4.06728200	-0.53627700	22.14717500
C	3.57891700	0.00259400	23.34807500
H	3.71910000	-0.56234900	24.26432500
C	2.91778100	1.24081800	23.41813800
C	2.76626900	1.96355800	22.22547700
H	2.27017800	2.93766800	22.23845100
C	4.80738400	-1.88523700	22.07672600
H	5.48113000	-1.94358900	24.17018600
C	2.37158500	1.81658400	24.73880100
C	0.84156800	1.99543100	24.61191500
H	0.57426000	2.69147800	23.80180400
H	0.42492700	2.39952700	25.54835200
H	0.34783500	1.03275500	24.40608400
C	3.03225400	3.18494700	25.02253600
H	4.12232400	3.08843800	25.13402400
H	2.63490600	3.61166900	25.95685200
H	2.83824400	3.91426400	24.21991700
C	2.65561000	0.89503200	25.93795200
H	2.18079300	-0.09132100	25.82019800
H	2.25121800	1.34739400	26.85611000
H	3.73543100	0.74539500	26.09301100
O	8.06789300	12.11251900	16.73434700
O	9.85188200	9.63652300	22.92936600
O	8.67007400	0.79399500	22.74796000
O	10.60105500	3.57434900	16.69675500
N	8.69248400	11.33500600	18.80338100
N	9.28094900	10.50709500	20.86929300
N	9.30856700	1.66890900	20.71251800
N	9.93029500	2.59505400	18.69260000
C	8.67256300	12.30773900	17.92959600
C	9.25944300	13.59064800	18.13566100
H	9.18609800	14.34569100	17.35257800
C	9.91572000	13.80481700	19.32395200
H	10.40001800	14.76364700	19.52748500

C	9.96998900	12.77026300	20.29857400	C	11.13420200	0.62842000	19.48459000
C	10.63429600	12.86598100	21.55125900	C	10.12422700	1.63135600	19.62800900
H	11.15940500	13.78770800	21.81562500	C	11.97784200	0.70186700	18.34321200
C	10.60241800	11.80277700	22.42251500	H	12.76856200	-0.04036900	18.20592300
H	11.08574800	11.82300100	23.39949900	C	11.78934000	1.70403000	17.42446200
C	9.87764600	10.64139000	22.02404100	H	12.40430900	1.81368600	16.53103700
C	9.30945500	11.53817800	19.99441700	C	10.72228600	2.62531900	17.64906000
C	8.86518200	8.67209300	23.01957000	C	9.48386800	4.35296200	16.45107800
C	9.23630900	7.52940900	23.74602700	C	9.75194400	5.57313800	15.80940300
H	10.26518200	7.43918400	24.09934700	H	10.78823100	5.87308500	15.64102100
C	8.27510800	6.57122500	24.03592300	C	8.68419000	6.36189500	15.40051600
C	6.94547400	6.73303300	23.60325300	C	7.35688500	5.97082500	15.66458900
C	6.59857400	7.84942700	22.85182900	C	7.10826400	4.76348700	16.30538300
H	5.56828500	7.98688000	22.51152900	H	6.08069700	4.44265200	16.49745600
C	7.55647300	8.82835000	22.55080100	C	8.17300600	3.93648600	16.69134800
H	7.28064900	9.70195000	21.96598800	H	7.98220600	2.97885400	17.16806700
C	7.49205200	5.45611400	26.05189700	C	7.94233900	7.40160900	13.32683000
C	6.15944800	5.62788100	25.62952200	C	8.37944200	7.51263300	12.01134100
C	5.13744800	5.76642600	26.56263500	H	9.41077300	7.80336900	11.79477700
H	4.10391100	5.89916700	26.23258700	C	7.48101200	7.24674900	10.96585400
C	5.44877800	5.73538400	27.93133700	H	7.81578100	7.33177900	9.92921900
H	4.65236300	5.84559100	28.67141200	C	6.16381700	6.87533100	11.24435700
C	6.77039100	5.56685900	28.35028000	H	5.47044700	6.67113900	10.42484900
H	7.00537800	5.54491100	29.41716500	C	5.72396900	6.76331800	12.57298100
C	7.80134800	5.42582000	27.40754000	H	4.69271300	6.47286700	12.79144100
H	8.83602600	5.29442000	27.73513900	C	6.61452100	7.02691700	13.60806300
C	8.47623600	5.31815900	24.88507100	C	6.32545100	6.95075700	15.11054700
H	9.51402800	5.18640500	25.21587700	H	5.29153100	6.65374000	15.33002300
C	7.96009100	4.14331800	24.05801100	C	8.77199100	7.65456100	14.58930900
C	6.62007100	4.30611100	23.65054100	H	9.80466500	7.95000600	14.36783100
C	6.01634800	5.62986900	24.10511300	C	8.00087600	8.71447700	15.37051400
H	4.97737600	5.75953800	23.77550200	C	6.67892700	8.32925400	15.66760800
C	5.96898700	3.28854200	22.96755200	C	5.83583900	9.20067800	16.35186000
H	4.92663900	3.40071000	22.67146100	H	4.80518000	8.91062800	16.57359400
C	6.65205600	2.10250000	22.66391100	C	6.30373600	10.46651400	16.73717100
H	6.15593600	1.28975800	22.13795300	H	5.66413500	11.17056200	17.27155200
C	7.98413600	1.97233400	23.03514300	C	7.60805100	10.83672700	16.42099700
C	8.65305800	2.98161200	23.74181400	C	8.46939400	9.96962000	15.73919800
H	9.69277300	2.83325300	24.04045900	H	9.48159400	10.29869000	15.49865800
C	9.44714000	0.75163400	21.63766500	C	6.24024400	-1.65378800	21.53958500
C	10.37695300	-0.32484700	21.57365800	H	6.23739900	-1.18774800	20.54137200
H	10.40755800	-1.05382500	22.38364300	H	6.77092600	-2.61505500	21.44906800
C	11.22423300	-0.36887300	20.49259800	H	6.82450800	-1.00848500	22.21354400
H	11.97504700	-1.15862600	20.40446300	C	4.91769200	-2.56176600	23.45404500

H	5.45083600	-3.51955800	23.35528800
H	3.92819600	-2.77862900	23.88545500
C	4.04421100	-2.83447700	21.12557700
H	3.01783200	-3.01006700	21.48413800
H	4.55630400	-3.80802200	21.06578900
H	3.97959400	-2.43202200	20.10288400
C	10.08898300	7.39683200	19.54170800
O	11.27663000	8.02801600	19.42807800

H@G3 (major isomer, PR3b)

O	22.73443700	17.24218300	29.77190900
N	21.67894100	18.95157700	22.14796800
C	23.64777700	22.28739300	19.82256500
C	22.75023700	21.67284400	20.70022000
H	22.68673300	21.99077800	21.74185500
C	21.93226200	20.61651100	20.27208300
C	21.98973200	20.17764300	18.94805600
H	21.34339900	19.35349800	18.63906400
C	22.85630800	20.78886900	18.02391500
C	23.67749200	21.82371800	18.49235200
H	24.36738600	22.29961500	17.79964700
C	24.62165600	23.38009700	20.29890400
C	24.11396000	24.07880100	21.57572300
H	24.81058700	24.88067600	21.86491000
H	24.04225900	23.39507900	22.43367000
H	23.12303200	24.53238100	21.41539400
C	25.97360900	22.69547200	20.60361100
H	26.37640700	22.19572700	19.70875400
H	25.85713300	21.93701800	21.39172200
H	26.71622700	23.43281500	20.94906900
C	24.82541900	24.45989700	19.21570400
H	25.47856800	25.25882800	19.59984600
H	23.86648000	24.91474900	18.92161900
H	25.30634500	24.06215900	18.31003000
C	22.83511800	20.35236500	16.54728600
C	23.98080500	20.98230400	15.73592600
H	23.94392500	20.62202700	14.69643000
H	24.96651600	20.71398600	16.14804900
H	23.90846500	22.08004100	15.70177900
C	21.48875500	20.79970600	15.93115100
H	21.43075700	20.50032800	14.87198200
H	21.37564600	21.89387000	15.98471100
H	20.63517900	20.34614400	16.45896700
C	22.95309600	18.81646400	16.43908100
H	22.09919900	18.30275200	16.90462600
H	23.87853300	18.45619500	16.91701000
H	22.97668000	18.51100900	15.38115500
C	21.01265400	19.94862500	21.25151100
H	20.55312700	20.68949600	21.92274900
H	20.20661100	19.40116500	20.74983100
C	23.00964900	18.70505300	22.11346200
H	23.58139800	19.21547100	21.34134900
C	23.61440500	17.88112700	23.04082500
H	24.69582200	17.75984600	22.97779400
C	22.84763100	17.28706100	24.06565300
C	21.45088500	17.49922800	24.00940000
H	20.77664000	17.08401500	24.75476700
C	20.89470000	18.32269500	23.05828500
H	19.81909000	18.50993700	23.00791500
C	23.45579500	16.59655300	25.20653900
C	24.79150800	16.86441300	25.57802000
H	25.42795200	17.47287300	24.93352300
C	25.28404000	16.45335500	26.80936400
H	26.29618000	16.70985600	27.12760600
C	23.16210200	15.39085300	27.31264000
H	22.51351000	14.82113300	27.97589600
C	22.67936700	15.80842100	26.08042300
H	21.65932200	15.53227500	25.80793000
C	24.15964900	15.32938400	30.07843100
H	23.30001500	14.66971300	29.87508300
H	24.79575600	14.82826000	30.82229800
C	23.68691100	16.65816900	30.64474800
H	24.57037500	17.31075100	30.77533100
H	23.25545100	16.46686600	31.64792800
O	28.41993600	17.50518800	22.25220800
O	24.03301000	22.11354900	24.98429300
O	16.11414700	18.69881000	24.13526500
O	20.27163000	16.95075900	18.91141800
N	26.96388000	19.07005600	23.11203300
N	25.49899700	20.61166300	24.01090700
N	17.48639600	18.21692000	22.34984500
N	18.85658100	17.62498100	20.59713100
C	28.15748700	18.76366300	22.66727400
C	29.25099500	19.67530500	22.57977700
C	29.03414800	20.96952600	22.97930100
H	29.83397000	21.71287500	22.92683500
C	27.75490300	21.34763900	23.47106000

C	27.42397700	22.65246300	23.91863000
H	28.17606000	23.44462300	23.87681400
C	26.16686000	22.90212900	24.41430300
H	25.85911200	23.87814700	24.78918500
C	25.24798500	21.81648600	24.45681300
C	26.73569900	20.34234100	23.52684300
C	23.24601600	21.20278200	25.66811600
C	21.89741100	21.57194100	25.80609000
H	21.55379000	22.50738000	25.35956100
C	21.05091600	20.75526000	26.54481700
C	21.52213600	19.55217400	27.10424200
C	22.86653000	19.22220700	27.00231300
H	23.24496700	18.31997800	27.48217400
C	23.73997200	20.05051300	26.28505200
H	24.78806200	19.77826000	26.19720300
C	20.43857800	18.75622200	27.83088500
H	20.81031800	17.80605300	28.23261800
C	19.58156200	21.01788900	26.86851300
H	19.21606100	21.97125200	26.46653700
C	19.46137200	20.92851000	28.39422200
C	18.97087500	21.90989700	29.24908700
H	18.61233400	22.86243700	28.84982300
C	18.94381900	21.66589300	30.63204900
H	18.55997100	22.43279800	31.30908500
C	19.40478600	20.45151700	31.14559300
H	19.37869800	20.27088800	32.22303800
C	19.89729800	19.45879900	30.28280600
H	20.25911500	18.50367800	30.67451700
C	19.92065700	19.70218500	28.91304700
C	18.82928300	19.81307600	26.31624000
C	17.78917000	19.84946900	25.39622900
H	17.41310300	20.79013300	24.98908500
C	17.20341300	18.64275800	25.00016800
C	17.63850800	17.41990500	25.50657600
H	17.15072600	16.50050600	25.17908700
C	18.69121500	17.39556400	26.43558700
H	19.03208400	16.44232100	26.84860300
C	19.29013800	18.58735500	26.83657600
C	16.29330400	18.49244100	22.81404200
C	15.11690800	18.59433600	22.01115700
H	14.16723900	18.83585100	22.48917500
C	15.24203200	18.38230600	20.66111500
H	14.37322800	18.45391900	20.00158600
C	16.51475400	18.05172200	20.11674000
C	17.61895200	17.96887500	21.02272900
C	16.76566100	17.78108100	18.74575600
H	15.94623600	17.84545400	18.02501200
C	18.02945300	17.41522500	18.33840800
H	18.27112400	17.17001300	17.30410500
C	19.03987800	17.33075400	19.33441400
C	21.15537900	16.22127400	19.69993800
C	22.48891900	16.24343900	19.26542200
H	22.76492900	16.85993900	18.41130700
C	23.41956600	15.43980800	19.90950800
C	23.04445100	14.63229300	20.99963900
C	21.72527600	14.63684500	21.43510500
H	21.41988600	14.00983000	22.27670200
C	20.76731400	15.42787300	20.78394300
H	19.73528300	15.41564100	21.12513800
C	24.19306800	13.78214600	21.53758000
H	23.89892300	13.15405100	22.38797900
C	24.88609100	15.27357400	19.52386400
H	25.18004200	15.90394200	18.67495500
C	25.07081700	13.77839600	19.25001900
C	25.55039900	13.20293600	18.07820100
H	25.84184000	13.83190300	17.23275900
C	25.65662100	11.80567900	17.99447900
H	26.03287800	11.34474400	17.07802200
C	25.28491900	11.00376600	19.07598600
H	25.37103600	9.91689000	19.00333000
C	24.80093900	11.58597900	20.25816000
H	24.51026500	10.95870200	21.10508900
C	24.69555100	12.97037800	20.34025900
C	25.32208800	14.75132900	21.87813500
C	25.99154900	14.86192000	23.09188700
H	25.71064700	14.23330500	23.93983800
C	27.03196600	15.79325800	23.22662900
H	27.57376700	15.90476000	24.16676100
C	27.37016400	16.59942700	22.14457600
C	26.71015400	16.49695100	20.91433300
H	27.01367000	17.13773000	20.08410800
C	25.69005300	15.56426900	20.78719400
H	22.63776400	18.18055800	29.98284200
H	30.20863100	19.32190300	22.19681000
C	24.45646700	15.76487100	27.71610100
O	24.98663600	15.49824600	28.92929800

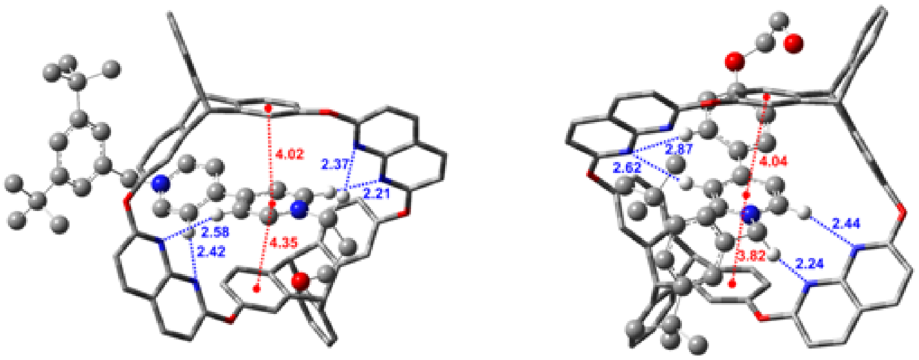

PR1a PR3b

Fig. S46 DFT optimized structures of **PR1a** and **PR3b**. The key distances for H-bonding interactions between N and H atoms (dash lines in blue) and π-π stacking interactions between pyridyl and phenyl ring centers (dash lines in red) are labeled in Angstrom. H atoms not involved in H-bonding are omitted for clarity.

11. References

[1] R. F. Munhá, L. G. Alves, N. Maulide, M. T. Duarte, I. E. Markó, M. D. Fryzuk and A. M. Martins, *Inorg. Chem. Commun.*, 2008, **44**, 1174-1176.

[2] A. Grégout, J. L. Delgado, J.-F. Nierengarten, B. Delavaux-Nicot, A. Listorti, C. Chiorboli and A. Belbakra, N. Armaroli, *New J. Chem.*, 2009, **33**, 2174-2182.

[3] All the units of the NMR spectra in the supporting information are ppm.

[4] In order that the ratio of the isomers could be calculated as precisely as possible, we did not make the effort to further purify the mixture of them, since the two integrated peaks chosen for comparison would not be affected by the small amount of remaining impurities.

[5] The relatively high value of *R* was probably caused by the high intrinsic nonsymmetry of the rotaxane **R3b** molecule itself.

[6] Gaussian 09, revision C.01; M. J. Frisch, G. W. Trucks, H. B. Schlegel, G. E. Scuseria, M. A. Robb, J. R.Cheeseman, G. Scalmani, V. Barone, B. Mennucci, G. A. Petersson, H. Nakatsuji, M. Caricato, X. Li, H. P. Hratchian, A. F. Izmaylov, J. Bloino, G. Zheng, J. L. Sonnenberg, M. Hada, M. Ehara, K. Toyota, R. Fukuda, J. Hasegawa, M. Ishida, T. Nakajima, Y. Honda, O. Kitao, H. Nakai, T. Vreven, J. A.Montgomery, Jr., J. E. Peralta, F. Ogliaro, M. Bearpark, J. J. Heyd, E.Brothers, K. N. Kudin, V. N.Staroverov, R. Kobayashi, J. Normand, K. Raghavachari, A. Rendell, J. C. Burant, S. S. Iyengar, J. Tomasi, M. Cossi, N. Rega, J. M. Millam, M. Klene, J. E. Knox, J. B. Cross, V. Bakken, C. Adamo, J. Jaramillo, R. Gomperts, R. E. Stratmann, O. Yazyev, A. J. Austin, R. Cammi, C. Pomelli, J. W. Ochterski, R. L. Martin, K. Morokuma, V. G. Zakrzewski, G. A. Voth, P. Salvador, J. J. Dannenberg, S. Dapprich, A. D. Daniels, O. Farkas, J. B. Foresman, J. V. Ortiz, J. Cioslowski, and D. J. Fox, Gaussian, Inc., Wallingford CT, 2009.

[7] a) A. D. Becke, *Phys. Rev. A*, 1988, **38**, 3098-3100; b) C. Lee, W. Yang and R. G. Parr, *Phys. Rev. B*, 1988, **37**, 785-789; c) A. D. Becke, *J. Chem. Phys.*, 1993, **98**, 5648-5652; d) A. D. Becke, *J. Chem. Phys.*, 1993, **98**, 1372-1377; e) P. J.Stephens, F. J. Devlin, M. J. Frisch and C. F. Chabalowski, *J. Phys. Chem.*, 1994, **98**, 11623-11627.

[8] F. Weigend and R. Ahlrichs, *Phys. Chem. Chem. Phys.*, 2005, **7**, 3297-3305.

[9] S. Grimme, J. Antony, S. Ehrlich and H. Krieg, *J. Chem. Phys.*, 2010, **132**, 154104.

[10] A. V. Marenich, C. J. Cramer and D. G. Truhlar, *J. Phys. Chem. B*, 2009, **113**, 6378-6396.
